# Knowledge Structure and Evolution of Global Ancient Tree Research: A Visual Exploration Based on CiteSpace

**DOI:** 10.3390/plants15203182

**Published:** 2026-10-17

**Authors:** Chaobin Xu, Yunni Chang, Baoyin Li, Quanlin Zhong, Sunbowen Zhang

**Affiliations:** 1 School of Geographical Sciences, Fujian Normal University, Fuzhou 350117, China; 2 Tea Research Institute, Fujian Academy of Agricultural Sciences, Fuzhou 350012, China

**Keywords:** heritage tree, global, web of science, CiteSpace, bibliometrics

## Abstract

Ancient trees, as rare natural and cultural heritage, are a global research focus. Based on 462 publications from the Web of Science Core Collection (1998–2025), covering 68 countries/regions and 403 institutions, our analysis shows the following: (1) Publication output grew steadily, with increasing interdisciplinary integration around growth and conservation. (2) China leads with 120 publications (25.97% of the total), followed by the United States with 75 (16.24%); together they account for 42.21%. The Chinese Academy of Sciences is a key contributor, but international collaboration remains limited. (3) Keyword analysis highlights conservation, growth, biodiversity, diversity, and forest as core themes, with recent bursts in biodiversity (5.39), climate (3.85), and forest management (3.47). Future trends include holistic conservation frameworks, climate response, belowground ecology, longevity mechanisms, and big data, digital twins, and AI for precise, preventive management. This work matters because ancient trees are not just old plants—they are vital parts of our shared natural and cultural heritage that benefit ecosystems and communities worldwide.

## 1. Introduction

Ancient trees serve as ecological cornerstones and cultural heritage [1]. Definitions of ancient trees vary across regions, species, and studies. For example, the Regulations on the Protection of Old and Famous Trees issued by the National Forestry and Grassland Administration of China define old and famous trees as woody individuals older than 100 years [2,3]. In this study, we follow this latter perspective and use a more restrictive operational definition: ancient trees are trees older than 150 years that have persisted through long-term environmental change and possess significant scientific, historical, or cultural value [4,5,6]. This 150-year threshold is not a universally accepted standard; it is adopted here for comparability with key international studies and to focus on long-lived individuals with pronounced ecological and heritage value. As representative plant species of regional development, ancient trees have endured long-term natural environmental changes. Their related physiological and biochemical indicators provide high-resolution natural evidence for studying regional and global changes as well as human activities [7,8]. At the same time, large ancient trees play a distinctive role in delivering ecosystem services such as carbon sequestration and sink enhancement, microclimate improvement, soil water supply capacity, and biodiversity enrichment [9,10]. They represent valuable forest genetic resources worthy of research and conservation [11,12]. Furthermore, ancient trees bear witness to historical changes and societal development [13]. However, due to factors such as species, age, health status, and environmental influences including temperature, precipitation, and pests, growth decline is emerging as these trees age [14]. Consequently, the conservation and management of ancient trees face unique challenges [15].

Ancient trees span long generation times and buffer environmental fluctuations, linking centennial environmental cycles. Their long-term ecological adaptability is crucial, while also providing valuable data on environmental history and individual lifespan [16]. Ecologically, ancient trees serve as anchor nodes for biodiversity and ecosystem complexity, enhancing overall forest stability and interactions. They play a key role in restoring the functions of disturbed ecosystems [17] and have attracted growing academic attention. Research on ancient and celebrated trees primarily covers their diversity, distribution patterns [18,19], conservation [20], value assessment [21], and relationships with environmental factors [22]. These studies have further advanced the field. However, existing research presents both strengths and shortcomings. For instance, current investigations often focus on isolated aspects while overlooking comprehensive developments and trends in the discipline. A thorough synthesis of the progress in ancient tree research would promote a more holistic understanding and expansion of this field.

Furthermore, existing bibliometric research has employed various methods and tools such as CiteSpace, VOSviewer, and HistCite to review research progress [23,24], thereby exploring more sub-fields, yet often overlooking “ancient trees” themselves. Thus, it is necessary to summarize research advances, current developments, key focuses, and potential trends, in order to gain a comprehensive understanding of “ancient trees,” grasp their overall status and characteristics, construct a complete knowledge framework for the field, assist researchers in selecting appropriate directions, and ensure their sustainable development. Although bibliometric methods have been applied to large old trees, reviews specifically dedicated to ancient trees remain scarce. Xie et al. provided an early overview (2000–2022) [25], but their broad LOT concept, limited temporal window, and conventional co-occurrence analysis left important gaps. A similar limitation appears in Chen et al., who compared Chinese and international old tree research using WoS and CNKI (1980–2019) and found that international studies emphasized physiological traits, specific species, and forest/ecological relationships, while Chinese studies focused on resource surveys, protection strategies, and rejuvenation techniques [26]. However, their work relied mainly on manual frequency statistics and narrative comparison rather than bibliometric mapping, did not isolate “ancient trees” from broader categories such as old trees and heritage trees, and ended in 2019. The present study therefore offers a stricter operational definition of ancient trees, updated coverage to 2025, finer-grained CiteSpace 6.4.R1 analyses, and an explicit diagnosis of knowledge fragmentation. These features make it more targeted, updated, and structurally informative than previous bibliometric reviews.

In summary, this study employs the CiteSpace visualization software to conduct a bibliometric analysis of research on ancient trees from multiple perspectives. Specifically, (1) a quantitative approach is adopted to systematically outline the academic landscape of the field, visualizing the evolution of research themes through collaboration network analysis, evaluation of academic impact, and identification of research hotspots and trends; (2) based on phenomena such as burst detection of keywords and integrating the aforementioned analyses, targeted future research directions are proposed, offering a roadmap for the sustainable development of related fields [27]. The study demonstrates innovation in the following three aspects: (1) it reviews the progress in ancient trees research from three dimensions—collaboration, co-citation, and co-occurrence—aiming to present a multidimensional and integrated knowledge landscape that helps readers grasp the dynamics of the field; (2) on a theoretical level, it attempts to construct a new knowledge framework to more clearly and intuitively reveal the internal logic and structural system of ancient trees research; (3) in practical terms, by highlighting current challenges and potential future directions, it seeks to raise sustained attention among academia and relevant stakeholders toward ancient trees research, thereby offering a useful reference for subsequent studies.

## 2. Literature Review

Ancient trees are revered as “living fossils” of human history and culture, having witnessed human settlement, development, and proliferation. They record changes in the local natural and geographical environment and represent key focal points for biodiversity conservation [28]. Simultaneously, they form integral components of regional ecosystems, embodying profound local cultural sentiments and spiritual sustenance, and hold significant social, ecological, cultural, and landscape value [29]. Research on ancient trees has progressively emerged as an interdisciplinary field spanning ecology, biology, geography, religious studies, and more [1,30,31], demonstrating a robust and synergistic academic landscape. This research primarily unfolds through diverse perspectives and evaluation methods.

### 2.1. Multidisciplinary Perspectives in Ancient Tree Research

Ancient tree research constitutes an interdisciplinary field characterized by a broad scope of perspectives, encompassing ecology, genetics, conservation biology, chronology, and cultural history. These diverse viewpoints aim to comprehensively elucidate the scientific value, ecological functions, and relationship of ancient trees with human society. From an ecological standpoint, studies focus on the pivotal role of these trees within ecosystems. This includes their function as keystone species supporting biodiversity, as well as their contributions to maintaining ecological balance and regulating microclimates [32]. Ancient trees often provide unique habitat structures that sustain rich biological communities, particularly in primary forest ecosystems. These complex structures facilitate extreme longevity and confer distinctive ecological characteristics [33]. The genetic perspective delves into the molecular and genetic foundations of longevity in ancient trees [34]. Research suggests that tree lifespan is not limited by a genetically determined senescence process; rather, longevity is achieved through specific life-history traits and resistance to or avoidance of external disturbances [35]. From a conservation biology angle, studies concentrate on assessing the health status of ancient trees and developing effective conservation and restoration strategies to address survival threats posed by global climate change and urbanization [36]. The cultural–historical perspective emphasizes ancient trees as composite carriers of cultural, historical, and social value [37]. They are often repositories of local legends, historical memory, and social customs, constituting vital elements of historical and cultural heritage. Integrating dendrochronological and socio-ecological research methods can elucidate the socio-ecological memory between people and trees [38].

### 2.2. Evaluation Methods in Ancient Tree Research

Regarding evaluation methods in ancient tree research, measurement techniques and models vary depending on the researcher’s focus. The existing literature on relevant methodologies can generally be categorized into chronological methods, geo-remote sensing methods, and big data/machine learning approaches. First, chronology, particularly dendrochronology, is a core method in ancient tree studies [39]. Analyzing tree-ring growth patterns enables the precise reconstruction of past climatic conditions, forest disturbance events, and the impact of human activities on the environment [40]. Stable isotope analysis of tree rings can capture climate trends and extreme events from inter-annual to millennial scales, though age-related biases in such measurements remain a topic of active research [41].

Concurrently, the increasing impact of climate change on forests—especially the negative effects of drought and extreme heatwaves on forest health and vitality—has prompted researchers to use remote sensing and dendrochronological data to assess forest vulnerability to climate change [42]. Furthermore, the application of modern analytical tools like R in ancient tree research has advanced data processing, analysis, and visualization, fostering the integration of innovative technologies such as multi-proxy datasets, artificial intelligence, and machine learning [43]. For instance, integrated digital twin models, combined with cyber-physical systems technology, utilize multi-source data and machine learning to evaluate the ecological environment quality of ancient trees and provide customized conservation strategies to enhance disaster response capabilities [33].

## 3. Materials and Methods

This study followed a transparent and reproducible bibliometric workflow using CiteSpace 6.4.R1. The workflow consisted of 4 steps: (1) data retrieval and cleaning; (2) CiteSpace parameterization and network construction; (3) network metrics, clustering, and burst detection; and (4) visualization, annotation, and interpretation.

### 3.1. Data Retrieval and Cleaning

**Figure 1:**
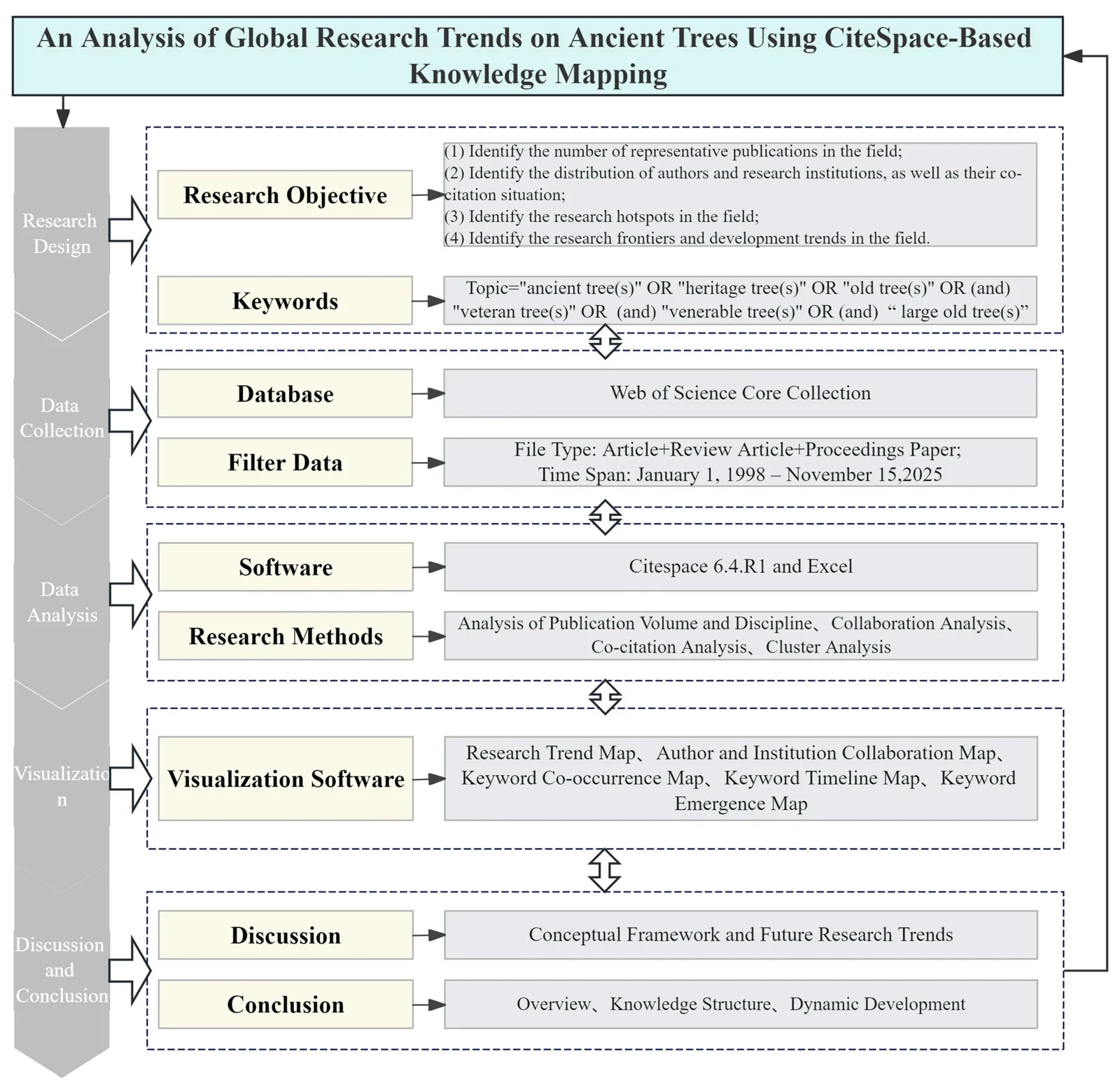
Technical roadmap.

Based on the Web of Science Core Collection database, an advanced search was conducted using the following topic search terms: “ancient tree(s)” OR “heritage tree(s)” OR “old tree(s)” OR (and) “veteran tree(s)” OR (and) “venerable tree(s)” OR (and) “ large old tree(s)” (Figure 1). The search was performed on 15 November 2025 and revealed that relevant publications primarily span the period from 1998 to 2025. A total of 477 related articles were identified in the Web of Science Core Collection. After removing document types such as Editorial Material, Meeting Abstract, and Letter, 462 publications in the field of ancient tree research remained for analysis. The search was limited to English-language publications.

Institution names were extracted from the C1 field of the WOS records. Before analysis, institution names were normalized and merged using the alias function in CiteSpace and manual checking. The normalization rules were as follows: (i) expanding standard abbreviations (e.g., Univ. → University; Acad. → Academy; Inst. → Institute); (ii) unifying capitalization, punctuation, and spelling variants; (iii) merging subordinate institutes, colleges, or laboratories into their parent university or academy when the parent–subunit relationship was explicit and the subunit was not an independent legal entity; (iv) using the latest official English name for renamed or merged institutions; and (v) retaining independent institutions as separate nodes. Two authors independently performed the normalization, and any discrepancies were resolved by discussion with a third author.

### 3.2. CiteSpace Parameterization and Network Construction

This study employs CiteSpace software (version 6.4.R1) developed by Professor Chaomei Chen for the visual analysis of data [44,45]. Using the data retrieved from the Web of Science Core Collection database, built-in calculation models of the software (e.g., for Country, Institution, Keyword) were applied to conduct co-occurrence and cluster analysis on the sample data. This process generated knowledge maps of the ancient trees field to identify its research hotspots. The specific analyses include the following: (1) Analysis of publication volume and subject categories: Publication trends and subject distribution maps were used to examine the annual changes in the number of publications related to ancient trees and their disciplinary affiliations, thereby analyzing research trends. (2) Collaboration analysis: Cooperation networks among research institutions and countries/regions were analyzed to understand collaborative relationships. (3) Citation analysis: Citation analysis of the literature, authors, and journals was conducted to explore research development and evolutionary dynamics. (4) Cluster analysis: Keyword co-occurrence maps were used to analyze main perspectives on the topic. Keyword timeline maps and burst detection maps were further examined to investigate research characteristics and hotspots [27].

The parameter settings for the visualization analysis are summarized in Table A1. The analysis covered January 1998 to December 2025, with a one-year time slice. Node types were country, institution, and keyword. For each time slice, nodes were selected using the g-index threshold with k = 25. The auxiliary selection parameters were LRF = 3.0, L/N = 10, LBY = 8, and e = 1.0. Link strength was calculated using the cosine function. To simplify the networks, both Minimum Spanning Tree (MST) and Pruning Sliced Networks were applied.

### 3.3. Network Metrics, Clustering, and Burst Detection

Betweenness centrality was used to measure node importance, calculated as

$$C_{B}(v)=\sum_{s\neq v\neq t}\frac{\sigma_{st}(v)}{\sigma_{st}}$$

where $\sigma_{st}$ is the number of shortest paths from node *s* to node *t*, and $\sigma_{st}(v)$ is the number of those shortest paths passing through node *v*. Centrality values were normalized to [0, 1], and nodes with centrality ≥ 0.1 were considered key bridge nodes. Clusters were labeled using the Log-Likelihood Ratio (LLR) algorithm. Modularity *Q* and silhouette *S* were used to evaluate clustering quality; *Q* > 0.3 and *S* > 0.5 were considered acceptable [42,43]. For keyword clustering, *Q* = 0.7724 and *S* = 0.8835. Burst detection was performed using Kleinberg’s algorithm.

### 3.4. Visualization, Annotation, and Interpretation

The visual outputs included publication trend and subject distribution maps, country and institution collaboration maps, journal and author citation charts, keyword co-occurrence networks, keyword timeline maps, keyword burst maps, and overall term timeline maps.

## 4. Research Findings

### 4.1. Publication Volume

**Figure 2:**
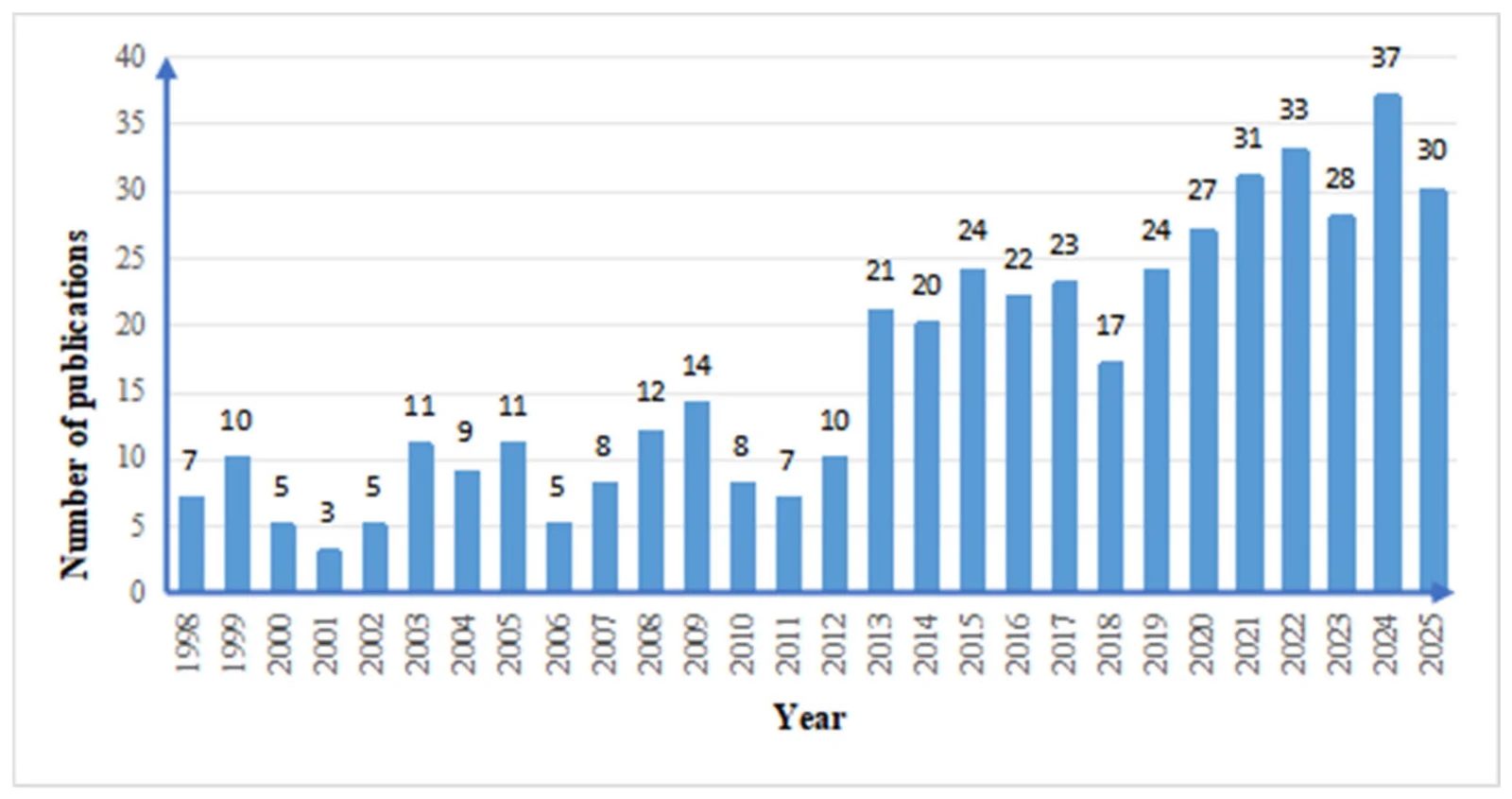
Trend of the number of published articles.

An analysis of the annual number of publications and its trends reflects the international development dynamics and academic interest in the field of ancient trees research. From 1998 to 2025, the overall publication output showed a fluctuating upward trend (Figure 2). Based on publication trends, the period can be divided into three phases: (1) The initial exploration phase from 1998 to 2012, characterized by a low and slowly increasing annual publication volume. During this time, research was still in its early stages, attracting limited academic attention, with theories and methods gradually being developed and refined. (2) The rapid development phase from 2013 to 2019. Following the declaration of March 21 as the “International Day of Forests” by the United Nations General Assembly in 2012 [46], the number of publications related to ancient trees grew at a relatively fast pace, albeit with minor fluctuations. In this phase, research topics expanded, methodologies became more diverse, interdisciplinary features began to emerge, and an overall research framework took shape. (3) The accelerated growth phase from 2020 to 2025, during which publication numbers increased significantly, reaching notably higher levels in recent years. This period saw a rapid rise in academic output, indicating widespread attention to the field, deepening research content, and increasingly close integration with practical applications. Overall, research in this area has continued to attract academic interest since the 1990s, with particularly rapid development over the past decade. The steady accumulation of research findings demonstrates strong disciplinary vitality and potential for growth. It is expected that this trend will continue in the future, playing an increasingly important role in both theoretical development and practical applications.

### 4.2. Analysis of Disciplinary Sources

**Figure 3:**
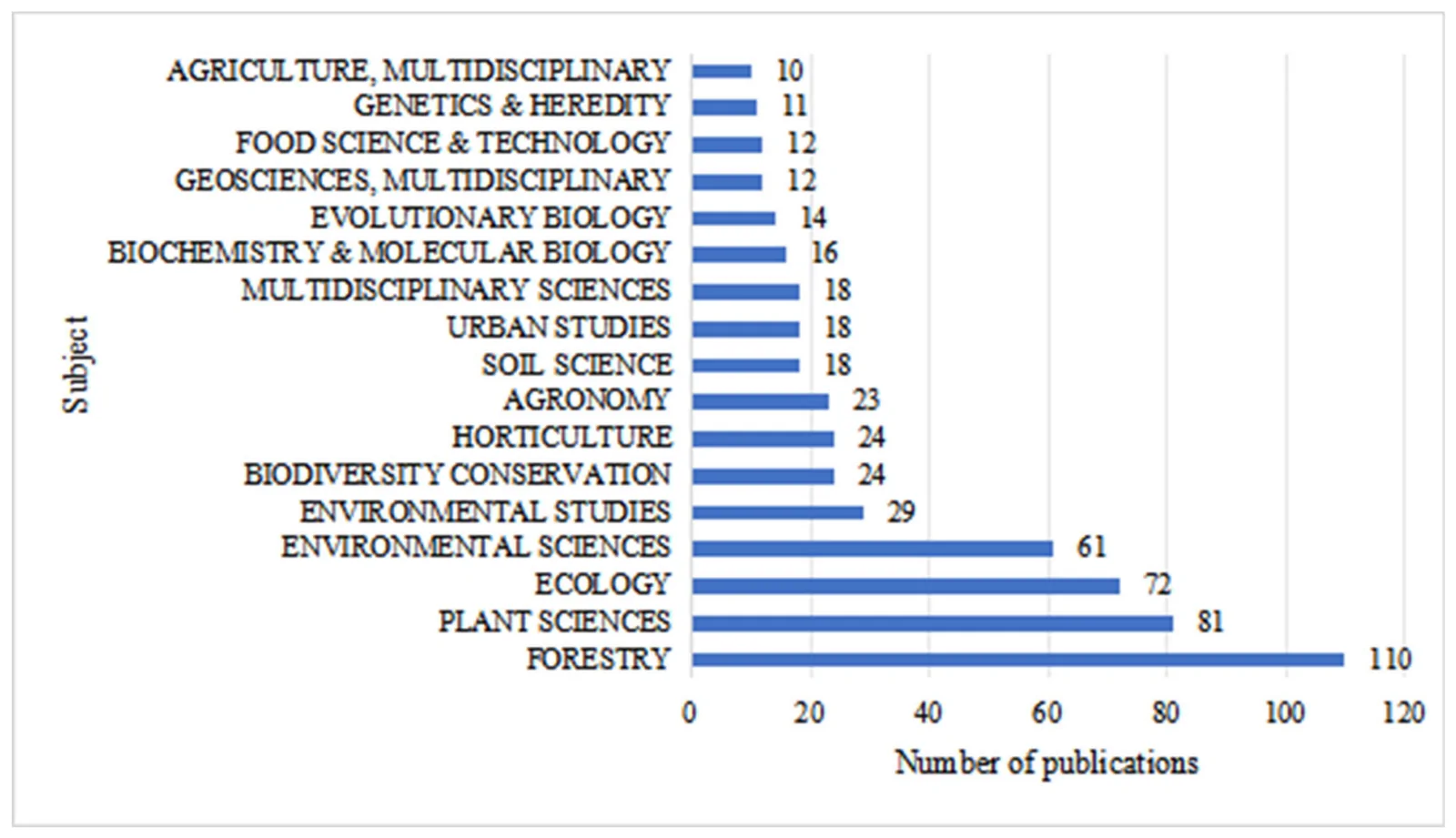
Distribution of disciplines.

The disciplinary distribution of research outputs and the number of article citations reflect the theoretical research value of related fields, providing direction for studies in the area of ancient trees [47]. In terms of research disciplines, ancient trees research primarily involves fields such as FORESTRY, PLANT SCIENCES, ECOLOGY, ENVIRONMENTAL SCIENCES, and ENVIRONMENTAL STUDIES (Figure 3). This indicates that, with the continuous advancement of research, interdisciplinary integration is driving the study of ancient trees from single processes toward multi-process synergistic development, and from local management to systematic regulation, making it a topic of shared interest across multiple disciplines. In the future, the field is likely to further integrate emerging disciplines such as EVOLUTIONARY BIOLOGY, contributing to more precise conservation strategies for ancient trees.

### 4.3. Analysis of Countries and Institutions

**Figure 4:**
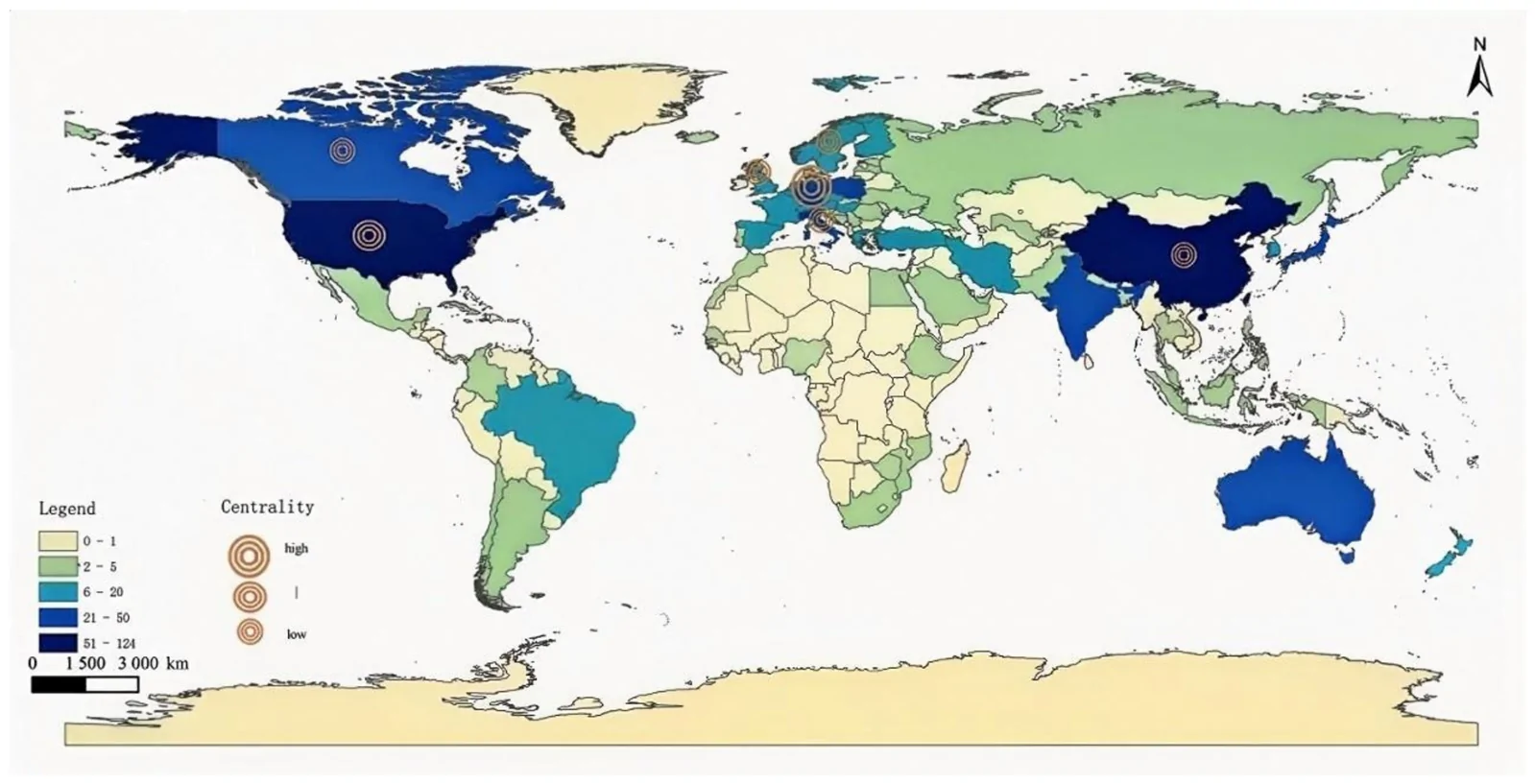
Distribution map of the number of published papers in various countries around the world.

An analysis of the national and institutional collaboration network in the field of ancient trees research globally from 1998 to 2025 covers 68 countries or regions and 403 institutions (see Figure 4). The top five countries by publication output are PEOPLES R CHINA, USA, GERMANY, INDIA, and CANADA. The top five countries by centrality are GERMANY, USA, PEOPLES R CHINA, CANADA, and SWEDEN. Among these, China and the United States have significantly higher publication numbers than other countries. China stands out as the most productive country (120 publications, centrality 0.19), accounting for 25.97% of the total output. The United States follows (75 publications, centrality 0.27), contributing 16.24% of the total publications. Together, these two countries account for 42.21% of all publications, indicating their central roles in international research.

**Figure 5:**
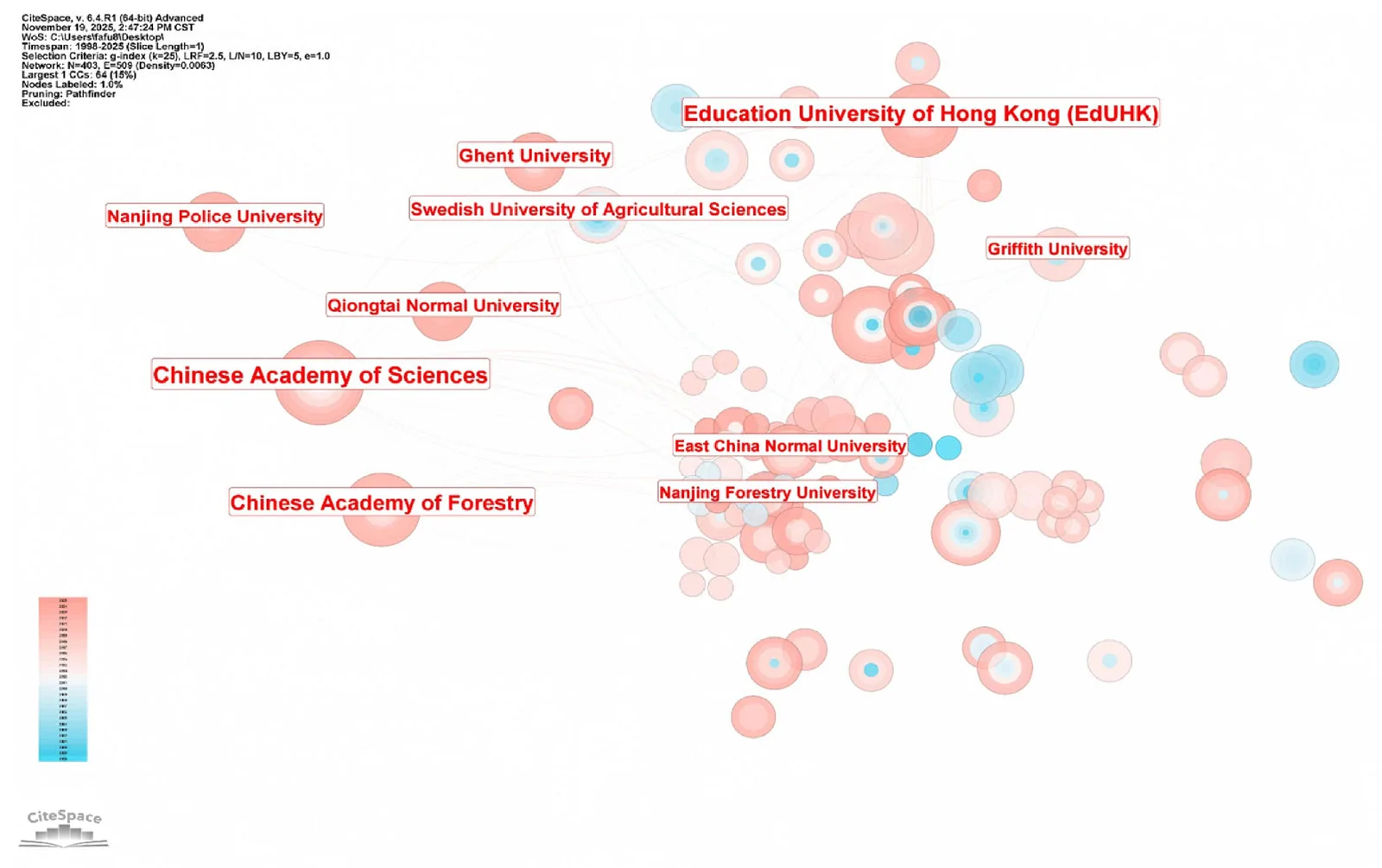
Institutional collaboration network in global ancient tree research, 1998–2025. Note: Nodes represent institutions; node size is proportional to publication frequency; node color encodes the time slice/year, with cool colors indicating earlier periods and warm colors indicating later periods; label size reflects frequency/centrality; links represent institutional collaboration, and link width indicates collaboration strength.

Based on the institutional collaboration co-occurrence map (Figure 5), the top five institutions in terms of publication output are Chinese Academy of Sciences, INRAE, Education University of Hong Kong, United States Department of Agriculture, and Chinese Academy of Forestry. The top five institutions by centrality are Chinese Academy of Sciences, INRAE, Education University of Hong Kong, United States Department of Agriculture, and Ghent University. The results show that China has a relatively large number of institutions engaged in research on ancient trees, with several ranking high on the list, reflecting the prominent performance of Chinese research institutions in terms of academic output in this field internationally.

Considering both country and institutional levels, China holds a notable advantage in publication volume, ranking third in country centrality, which indicates its substantial overall influence in this research area. With the exception of the Chinese Academy of Sciences, the centrality of other institutions is generally low (≤0.01), suggesting limited collaboration among them. There is a need to strengthen international cooperative efforts between institutions in this field.

**Figure 6:**
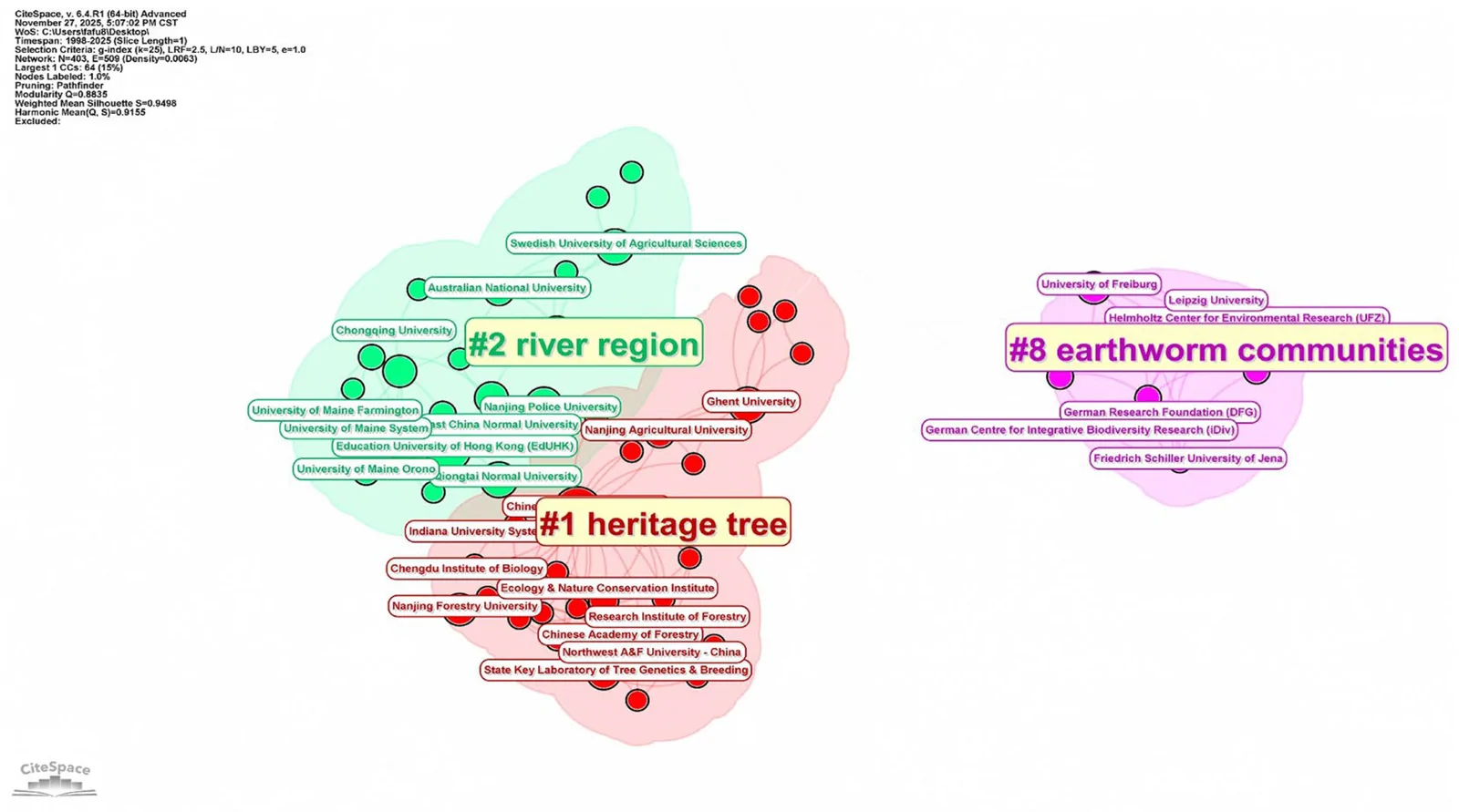
Cluster map of global research institutions in ancient tree research areas, 1998–2025. Note: Nodes represent institutions; node color denotes cluster membership; node size reflects publication frequency; links represent institutional collaboration; cluster labels are generated by the Log-Likelihood Ratio (LLR) algorithm.

Furthermore, this study conducted a cluster analysis of publishing institutions. The clustering results (Figure 6) indicate that research institutions in the field of ancient trees are mainly focused on three areas: river region, heritage tree, and earthworm communities.

(1) Regarding river region, the cluster brings together several universities from China and the United States, reflecting a collaborative network in river ecology, water resource management, and regional environmental studies. It includes branches of the University of Maine System in the U.S., as well as Chinese normal and agricultural/forestry universities such as Nanjing Agricultural University and Central China Normal University. This demonstrates the cross-regional and interdisciplinary nature of the field, likely centering on river ecosystem protection and watershed sustainable development.

(2) In the heritage tree cluster, research is centered around “heritage tree” and involves leading Chinese institutions engaged in forest genetics, forest conservation, and tree breeding, including the Chengdu Institute of Biology, Chinese Academy of Sciences; Nanjing Forestry University; the Chinese Academy of Forestry; and Northwest A&F University. The participation of the National Key Laboratory of Forest Genetics and Tree Breeding highlights the academic standing of this cluster in tree conservation and genetic improvement. This network concentrates on the protection of ancient and famous trees, forest germplasm resources, and forest ecological restoration.

(3) The earthworm communities cluster is primarily composed of German research institutions, such as the German Research Foundation (DFG), the German Centre for Integrative Biodiversity Research (iDiv), and the University of Jena. This indicates a strong research foundation in soil fauna ecology, biodiversity monitoring, and the role of earthworms in ecosystems, with particular emphasis on the ecological functions of earthworm communities and their application in biodiversity conservation.

### 4.4. Important Literature, Journals, and Scholars in the Field of Ancient Trees

In CiteSpace citation network analysis, the highly cited literature typically represents foundational or pioneering research with significant influence in the field. The co-cited literature, formed when documents are cited together, effectively reflects the knowledge base of a research area [48,49]. Therefore, conducting a co-citation analysis using CiteSpace in this study helps explore the ranking of the literature, journals, and authors with varying levels of impact.

#### 4.4.1. Highly Cited Literature

**Figure 7:**
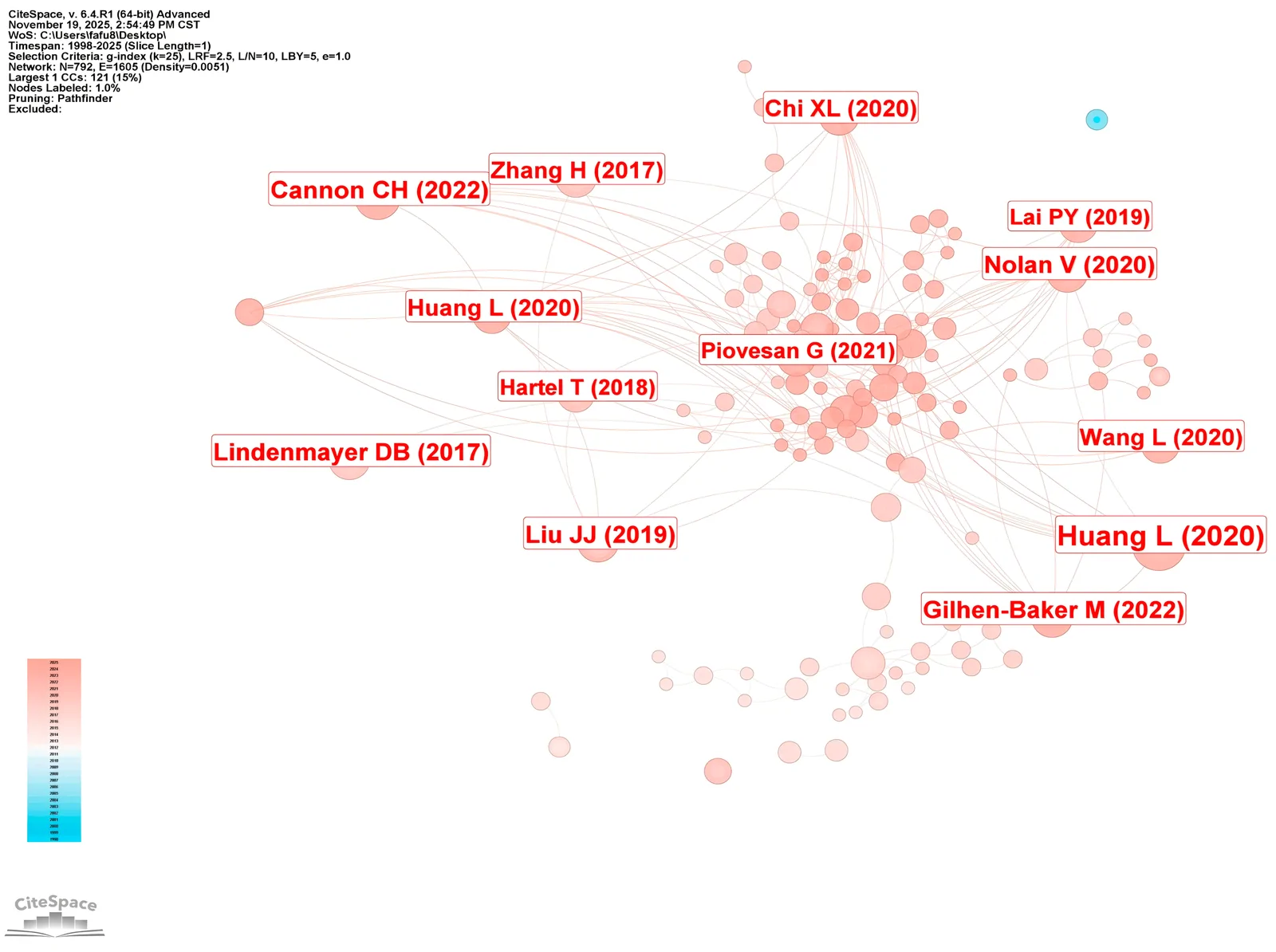
Top 10 most cited publications in ancient tree research.

Based on data from WOS, the ten most frequently cited publications in the field of ancient trees were compiled (Figure 7). The most cited work is by Huang et al. on the conservation of large ancient trees [9], with 16 citations, indicating that this study has attracted sustained academic attention in recent years and may serve as a key reference in the field. Additionally, publications by Cannon [50], Gilhen-Baker [51], Liu [18], Lindenmayer [52], and Nolan [53] each have citation counts exceeding ten, suggesting their high recognition and academic influence on related topics. It is noteworthy that all listed documents have a betweenness centrality of zero. At first glance, this indicates that although widely cited, they do not occupy “bridge” positions connecting different research clusters; in other words, they are not structurally critical intermediary nodes. More importantly, however, this pattern should be interpreted together with the low density of the bibliometric networks. For example, the keyword co-occurrence network contains 644 nodes and 1537 links but has a density of only 0.0074, and most institutions have centrality values ≤0.01. Such sparse connectivity suggests that the field is not merely lacking individual bridge nodes; rather, it exhibits a fragmented knowledge structure in which highly cited works function as local hubs within separate subfields but rarely link these subfields together. Citation flows are therefore concentrated within relatively independent thematic communities, and theoretical or methodological connections across ecology, genetics, microbiology, climate science, and social science remain underdeveloped. This fragmentation may constrain the emergence of integrated conservation frameworks and helps explain why the field, despite increasing publication volume and several highly cited studies, has not yet formed a tightly connected knowledge core. In terms of publication years, the highly cited literature mainly clusters between 2020 and 2022, with several works from 2020 showing particularly high citation frequencies. This indicates that this period may have been a peak phase for knowledge production in the field and highlights the rapid dissemination and concentrated academic focus on recent studies. Earlier works, such as those by Lindenmayer [52] and Zhang [54], continue to maintain high citation counts, demonstrating the lasting relevance of their research themes.

#### 4.4.2. Highly Cited Journals

Based on WOS data, Table 1 lists the 20 English-language journals with the highest citation counts in the field of ancient trees research. Among these, *Forest Ecology and Management*, *Biological Conservation*, *Nature*, *Science* and *PLOS One* are the top five most cited journals. Furthermore, according to the 2024 JCR classification, most of these journals are ranked in JCR Q1, representing leading publications in their respective fields. The exceptions are *PLOS One*, *Canadian Journal of Forest Research*, *Forests*, and *Oecologia*, which are categorized as JCR Q2. Therefore, for researchers in the ancient trees field, regularly following relevant articles in these journals can be an effective way to stay updated on the latest research developments.

#### 4.4.3. Highly Cited Authors

**Figure 8:**
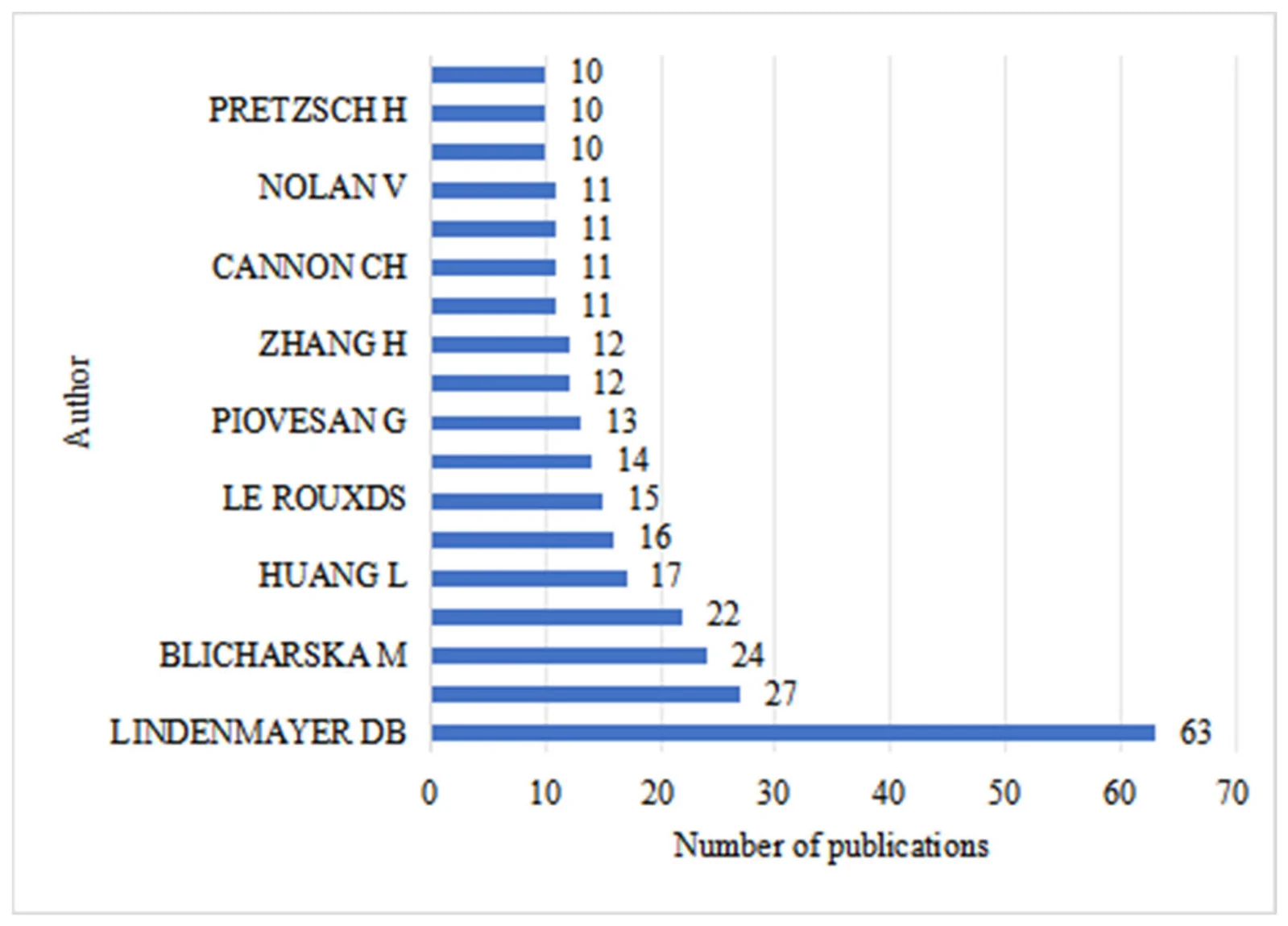
Most cited authors in ancient tree research.

As shown in Figure 8 among existing studies, the authors with more than 20 citations are, in descending order: Lindenmayer, David B. from Australian National University, whose work has been cited 63 times in total, making him the most cited researcher in the field to date and one of its most representative scholars. Next are Jim, C. Y. from the University of Hong Kong, with 27 citations; Blicharska, Malgorzata from Uppsala University, with 24 citations; and Liu, Jiajia from Fudan University, with 22 citations.

### 4.5. Trends in Keyword Characteristics in the Field of Ancient Trees

#### 4.5.1. Keyword Co-Occurrence

**Figure 9:**
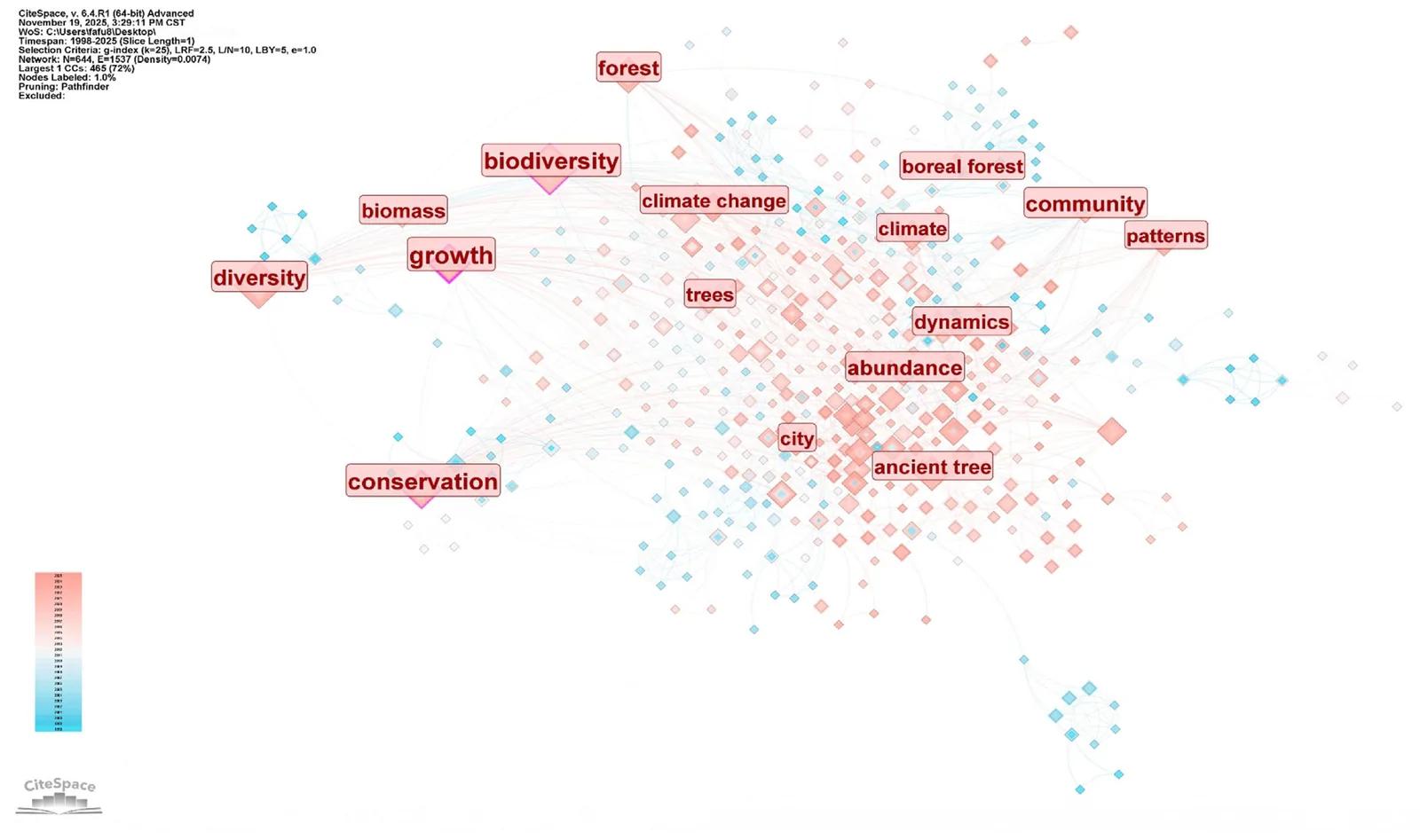
Keyword co-occurrence network of ancient tree research, 1998–2025. Note: Nodes represent keywords; node size is proportional to keyword occurrence frequency; label size reflects centrality; node color encodes the time slice/year, with cool colors for earlier periods and warm colors for later periods; links represent keyword co-occurrence, and link width indicates co-occurrence strength.

**Figure 10:**
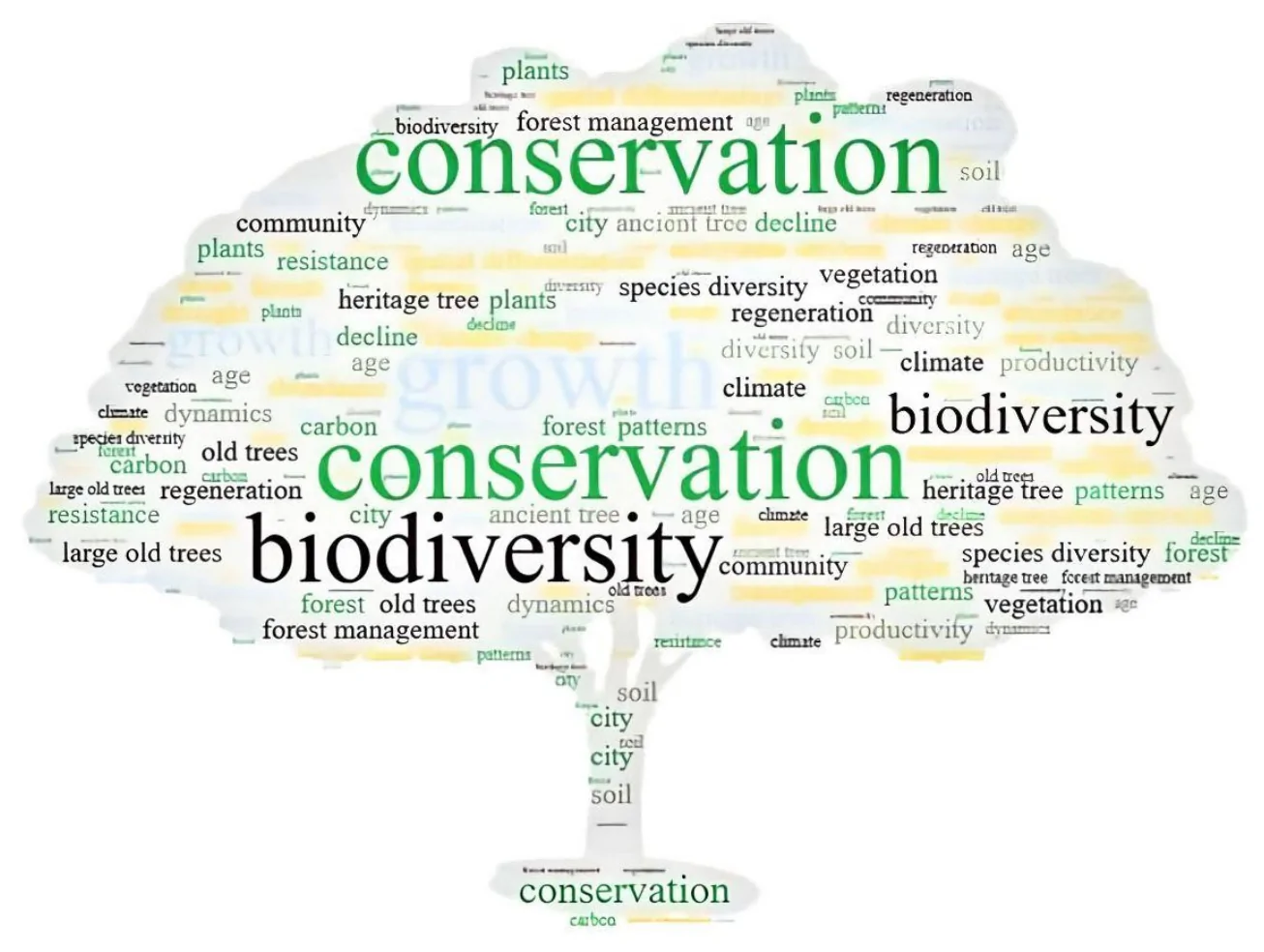
Frequency of the top keywords in ancient tree research, 1998–2025.

Keywords, as specialized terms reflecting the core content of the literature, can reveal research hotspots in a specific field through co-occurrence analysis [55]. This study employed Citespace 6.4.R1 to conduct keyword co-occurrence analysis on the relevant literature. The resulting co-occurrence network consists of 644 nodes and 1537 links, with a network density of 0.0074. In this map, node size corresponds to the frequency of keyword occurrence, node label size indicates the strength of centrality, and connecting lines represent co-occurrence relationships between keywords. After merging overlapping keywords and removing search terms, the results (Figure 9 and Figure 10) show that the five most frequent keywords are conservation, growth, biodiversity, diversity, and forest. The five keywords with the strongest centrality are growth, biodiversity, conservation, diversity, and biomass, each with a centrality value greater than or equal to 0.1. This suggests that although scholars in the field of ancient trees focus on different aspects, growth, biodiversity, conservation, diversity, and biomass serve as key nodes and are representative [56]. Furthermore, it can be observed that while the publication volume related to biomass may not be as high as that of other keywords, its high centrality underscores its importance as a critical node in ancient tree research, making it a key term for further studies in this area.

#### 4.5.2. Keyword Timeline

**Figure 11:**
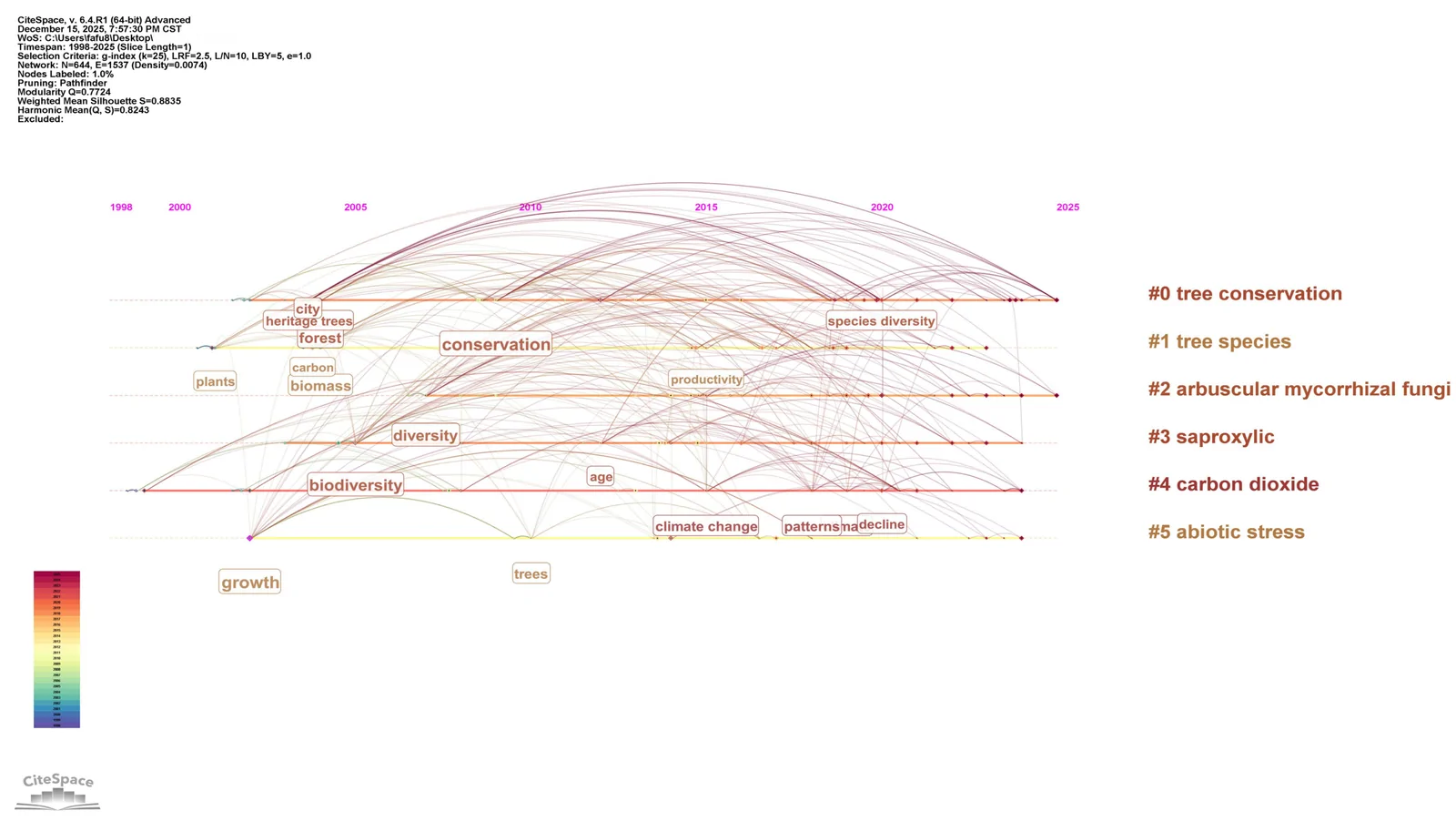
Keyword timeline chart.

This study employed the Log-Likelihood Ratio (LLR) method within CiteSpace software to analyze keyword trends over time. The results show a Modularity Q value of 0.7724 (>0.3) and a Silhouette value of 0.8835 (>0.5), indicating a significant and reasonably sound cluster structure [57]. A total of 16 clusters were generated. After removing duplicates and clusters containing fewer than 10 publications, six main clusters were retained (Figure 11).

#0 tree conservation: Key keywords include: trees; forests; Australia; birds; management factor; climate; range; scenario; bioclimatic. This cluster focuses on the impact of management factors on the conservation of ancient trees. It examines how different forest management strategies affect tree diversity, community composition, population structure, and carbon storage [58,59,60]. Large ancient trees hold critical importance for global biodiversity and are vital components of terrestrial ecosystems, warranting further research into their ecology, function, evolution, and management [61]. Furthermore, complex ecological relationships exist between birds and ancient trees, with large trees being a key factor for maintaining bird diversity in spruce-dominated production forests [62].

#1 tree species: Key keywords include: organic; trees; improved; fallows; intake gene; sequencing; treedivnet; plantation; its-rrna. This research direction emphasizes the use of advanced technologies to reveal the genetic diversity and ecological functions of ancient trees [63]. For instance, chromosome-level genome assembly techniques have been applied to sequence ancient species like Pinus strobilacea [64]. Studies utilize methods such as genome sequencing and ITS-rRNA analysis to gain deeper insights into the genetic structure and evolutionary history of ancient trees [65]. Ancient DNA analysis also offers new perspectives for understanding plant evolution and migration, as samples thousands of years old can still provide crucial allelic information. Revealing the ecological and genetic characteristics of ancient trees to promote their conservation and sustainable use has become a significant topic in ecology and genetics [66].

#2 arbuscular mycorrhizal fungi (AMF): Main keywords include: carbon; mixtures; sequestration; budget; pinus models; climate-growth; responsiveness; spruce; linear. Recent research increasingly focuses on the influence of AMF on the growth of ancient trees under climate change [31]. AMF can enhance plant growth and productivity by promoting the uptake of nutrients like phosphorus and nitrogen, with effects particularly pronounced under abiotic stress conditions [67]. Simultaneously, AMF participate in regulating the rhizosphere microenvironment and soil microbial community structure, affecting plant drought, salt, and heavy metal tolerance [68]. Their potential application in forestry management for restoring degraded ecosystems and improving soil health is also gaining attention [67]. Moreover, AMF can promote soil aggregate formation, contributing to the stabilization of soil carbon pools [69].

#3 saproxylic: Main keywords include: diversity; republic; sequestration; models; effects tropical; timber; defenses; reforestation; defoliation. The relevant literature uses dendrochronological methods to reconstruct the growth patterns and environmental interaction history of ancient trees [37,70]. Research also examines the ecological role of ancient trees in supporting saproxylic insect diversity, as these insects are essential for decomposing dead wood and nutrient cycling [71].

#4 carbon dioxide: Main keywords include: carbon; biomass; uptake; expansion; reforestation national; klamath; oregon; rule; fremont-winema. Publications concentrate on the role of ancient trees in biomass accumulation, carbon uptake expansion, and their function in reconstructing forest ecosystems [72], with specific studies in areas like Klamath [73] and Oregon [74]. By assessing the contribution of forest reforestation strategies to carbon sequestration [72] and the carbon uptake capacity of trees across different age classes [75], these studies explore how scientific conservation and management can enable sustainable use of ancient tree resources, thereby informing the optimization of global ecosystem services [76].

#5 abiotic stress: Main keywords include: leaf; rate; litterbag; nitrogen; specific shoot; axillary; citrifolia; capacity; induction. This group of the literature centers on the physiological and ecological response mechanisms of ancient trees to various abiotic stress conditions [77]. Additionally, research involves the regulatory role of root–shoot interactions in the plant’s overall response under abiotic stress [78]. Investigating the adaptation strategies of ancient trees to cope with abiotic stress has become a major focus in the global research field [79].

#### 4.5.3. Keyword Hotspots

Burst keywords refer to those experiencing a sudden increase in usage frequency within a specific timeframe [80]. They represent emerging hotspots and reflect the current research frontiers in a field [27]. A knowledge map visualization of burst keywords in the global “ancient trees” research domain from 1998 to 2025 was conducted using CiteSpace software. The analysis indicates that these are core focuses in current research and are likely to remain significant in the foreseeable future. Table 2 shows the following:

(1) The keyword “forest management” experienced a burst starting in 2016, remaining active until 2021 with a burst strength of 3.47. Forest management practices are crucial for the conservation and sustainable utilization of ancient trees [33]. The objectives of modern forest management have expanded beyond timber production to increasingly emphasize multifunctionality, encompassing ecosystem services such as carbon sequestration, biodiversity, and recreation [81]. The findings underscore its fundamental role in ancient tree research.

(2) The burst keyword “biodiversity” emerged in 2017, remaining active until 2023 with a high burst strength of 5.39, highlighting its importance in this field. Ancient trees serve as vital reservoirs of biodiversity, providing critical habitats and microhabitats for numerous organisms [82]. Forest biodiversity is essential for ecosystem health and delivers key services to humanity [83]. Furthermore, soil biodiversity is influenced by both living and dead trees, emphasizing the importance of forest characteristics for its structure, alongside regional climate and soil conditions [84].

(3) The burst keyword “climate” appeared in 2019 and remains active through 2025, with a burst strength of 3.85. As a major component of terrestrial ecosystems, forests play a key role in mitigating climate change but are themselves vulnerable to its impacts [85]. Increasing frequency and severity of climate-related disturbances may undermine past and ongoing efforts to use forests for climate mitigation and biodiversity conservation [86]. The results reflect the growing concern regarding the impacts of climate change on ancient trees and their ecosystems, as well as the role these trees play in climate regulation.

### 4.6. Temporal Trends of Overall Terms Including Keywords, Abstracts, and Titles in the Field of Ancient Trees

**Figure 12:**
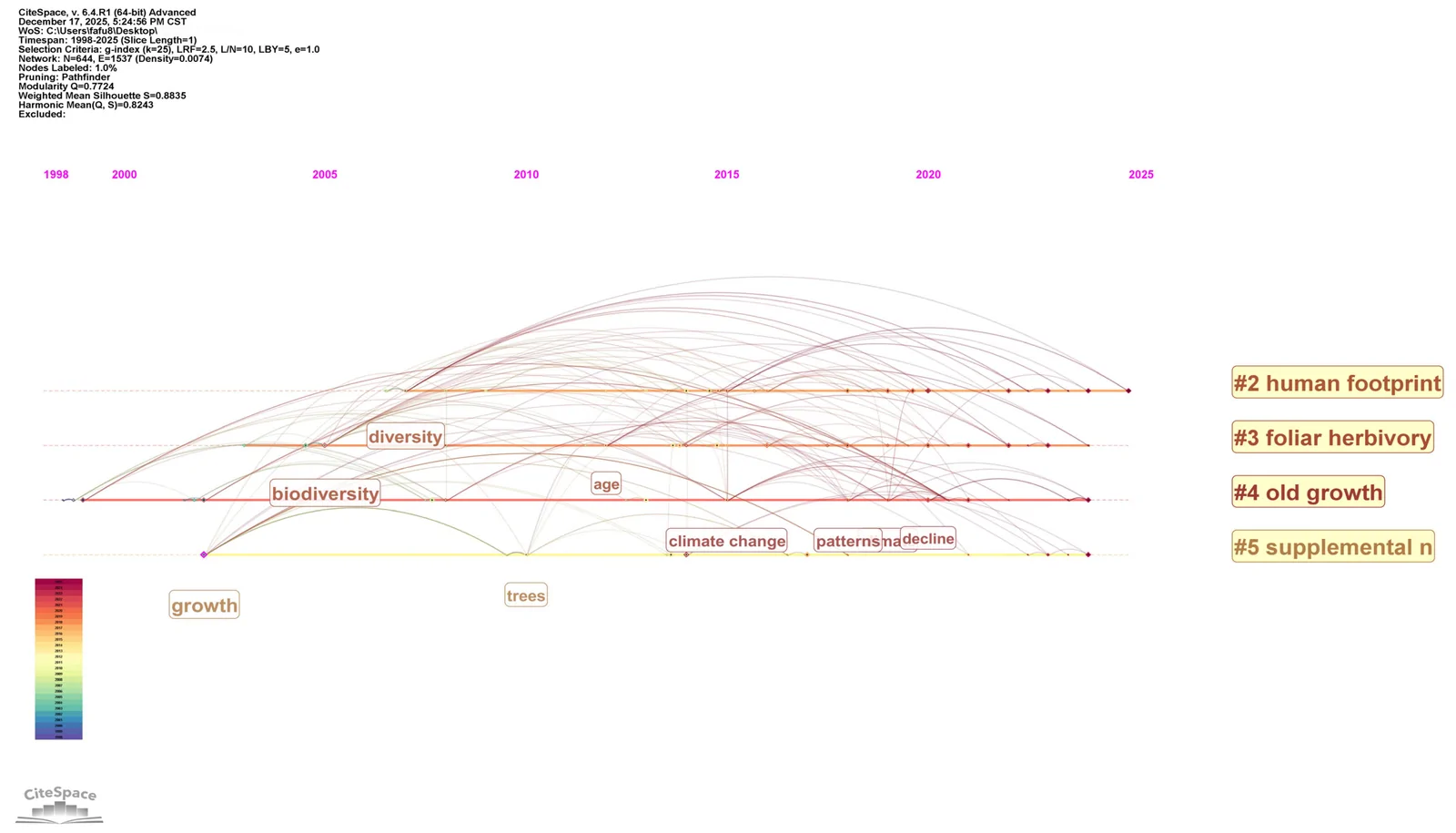
Overall word timeline chart.

To further identify key trends in the field of ancient trees, this study examines terms from keywords, abstracts, and titles. The temporal trend analysis of overall vocabulary was conducted using the Log-Likelihood Ratio (LLR) method in CiteSpace software. The results show a Modularity Q value of 0.7724 (>0.3) and a Silhouette value of 0.8835 (>0.5), indicating a significant and reasonably high clustering structure. The analysis generated 16 clusters in total. After removing duplicates and clusters with fewer than 10 publications, four main clusters were retained (Figure 12).

#2 Human Footprint: Keywords mainly include community; macrofauna; complex zone; tree-crop; agroforestry, etc. Large old trees contribute to ecosystem integrity and biodiversity in urban and suburban areas [61]. This cluster of the literature primarily focuses on the symbiotic relationship between humans and ancient trees. Initially, the perspective was mainly from natural science, concentrating on the effects of mature agroforestry systems on nodulation and yield, and the role of biological communities within them [87,88]. As research progressed, increasing scholarly attention turned to the socioeconomic value of agroforestry systems [87], gradually giving rise to research trends related to forestry heritage [89].

#3 Foliar Herbivory: Research on the impact of foliar herbivores on ancient trees is a multidimensional interdisciplinary field. It focuses on core concepts such as carbon sequestration, carbon stocks, species identity, species origin, annual dynamics, and protection strategies, particularly in biodiversity hotspots like China. Recent studies highlight that foliar herbivore activity can inhibit arbuscular mycorrhizal fungi (AMF) colonization by affecting symbiotic signals in plant root exudates [90]. Meanwhile, as crucial components of ecosystems, the carbon storage and sequestration capacity of ancient trees are key to climate change research [91]. Their conservation involves not only understanding their ecological functions but also studying the capacity of their ecosystems to resist external disturbances [92]. Therefore, a deeper understanding of the complex interactions between foliar herbivores and ancient trees is essential for developing effective conservation and carbon management strategies.

#4 Old Growth: Keywords mainly include canopy; resilience; patches; greenness; semiarid research; quantitative; country; monuments, etc. The canopy plays a vital role in ancient tree growth, influencing light conditions and microclimate, and is closely linked to biodiversity [93]. Research shows that the ecological resilience of old-growth forests in the face of climatic disturbances like drought has garnered significant attention [94]. Using remote sensing, researchers monitor changes in canopy greenness and moisture to assess the resilience of semiarid forests, finding associations between canopy greenness and structural complexity [95]. Furthermore, patch dynamics within ancient forests are crucial for understanding their structure and function [96]. For example, patch dynamics in longleaf pine savannas affect understory vegetation composition and spatial heterogeneity [97].

#5 Supplemental N (Nutrition/Features): Keywords mainly include patches; greenness; semiarid research; quantitative; country, etc. Ancient tree communities often exist as spatially heterogeneous patches, playing important roles in maintaining ecosystem stability and regulating hydrological processes [98]. The growth and recovery capacity of shrub patches can enhance the resilience of semiarid ecosystems, especially under prolonged human disturbance [99]. Additionally, research on ancient trees in semiarid regions frequently employs quantitative analysis to reveal their growth patterns, responses to climate change, and characteristics of soil carbon and nitrogen distribution [100].

## 5. Discussion

### 5.1. Research Prospect

#### 5.1.1. Ancient Trees Interaction from a Social–Ecological System Perspective

Future research on ancient trees will extend beyond traditional ecology, fostering deeper integration with cultural history, remote sensing science, sociology, and economics to establish a systematic conservation framework that integrates “nature–culture–society” as a unified whole [61]. Studies show that ancient trees are not only ecological entities but also carriers of cultural memory and social identity [27]. Future work should further examine how cultural spaces such as religious sites, historical relics, and traditional villages function as “social refuges” for ancient trees, and analyze the underlying institutional, belief-based, and community governance mechanisms. At the same time, interdisciplinary assessment tools should be developed to integrate ecological service value with cultural heritage value, providing a theoretical basis for conservation policies that balance ecological integrity, cultural sensitivity, and social sustainability [89,101]. Moreover, research should explore cultural and tourism initiatives centered on ancient trees in historic villages. A key trend is investigating how to translate the ecological and cultural worth of ancient tree communities into social recognition and economic benefits through sustainable livelihood and community-based enterprise models, such as eco-tourism, cultural heritage tourism, creative cultural products, and non-timber forest products. This would help establish long-term conservation mechanisms and ultimately promote the shift in ancient trees from “isolated objects of protection” to a “living heritage system” [89].

#### 5.1.2. Climate Change Response and Adaptive Management Research

With “climate” emerging as one of the most prominent keywords in recent years [102], the response mechanisms and adaptation strategies of ancient trees to climate change will become a core frontier. Future studies need to focus on revealing the physiological thresholds, resilience, and decline mechanisms of ancient trees under extreme climate events, with particular attention to differential responses across tree species, age structures, and habitat types [103]. In addition, ancient trees serve as invaluable archives for reconstructing past fire and precipitation regimes through dendrochronological analyses. Tree-ring fire scars and stable isotopes can provide centennial-to-millennial records of fire frequency, drought variability, and hydroclimatic shifts, although such reconstructions are species-dependent and require careful cross-dating [104]. Representative ancient tree species span diverse taxonomic and geographic contexts, including long-lived conifers such as Pinus longaeva, Sequoiadendron giganteum, and Juniperus spp. in North America and Eurasia; broadleaf species such as Quercus robur, Castanea sativa, and Olea europaea in Europe and the Mediterranean; Ficus spp. and Taxodium mucronatum in tropical and subtropical regions; Adansonia digitata in Africa; Eucalyptus spp. in Australia; and Araucaria araucana in South America. In China, common ancient tree species include Ginkgo biloba, Cinnamomum camphora, Phoebe zhennan, Cupressus funebris, Styphnolobium japonicum, and Gleditsia sinensis. These long-term records not only highlight the unique scientific value of ancient trees but also offer essential baselines for anticipating future climate–fire interactions. Methodologically, approaches such as tree-ring anatomy, stable isotope analysis, high-resolution remote sensing, and process-based models should be combined to build a multi-scale climate sensitivity assessment system spanning the “individual–community–landscape” levels [95]. On this basis, forward-looking adaptive and intelligent forestry management strategies should be developed, with their effectiveness validated through long-term monitoring networks, so as to enhance the survival capacity of ancient tree populations in the face of irreversible climatic shifts.

#### 5.1.3. Symbiotic Mechanisms and Ecosystem Functions of Ancient Trees

Research indicates that belowground ecological processes are crucial for the health and functioning of ancient trees [90]. Future studies should systematically elucidate the composition and function of the unique microbial communities in the rhizosphere of ancient trees and establish causal relationships with their longevity and stress resistance. Specifically, investigations could explore how arbuscular mycorrhizal fungi (AMF) regulate phosphorus acquisition efficiency, water use strategies, and the accumulation of secondary metabolites in ancient trees under low-nutrient stress. Concurrently, attention should be paid to the soil carbon and nitrogen cycling processes driven by litter and woody debris from ancient trees and their long-term effects on belowground biodiversity and forest carbon sink functionality. This will provide a scientific basis for developing soil health management technologies based on microbial regulation and early warning systems for the decline of ancient trees [67]. Furthermore, habitat restoration and ecological enhancement techniques are key to maintaining ancient tree vitality. Research on rhizosphere micro-ecological restoration is needed, utilizing microbiome engineering to improve soil physicochemical properties and biological activity, thereby promoting nutrient cycling and rebuilding disease-resistant symbiotic systems [88]. Additionally, addressing common issues such as restricted growing space, soil compaction, and changes in groundwater hydrology requires the development of low-interference physical restoration technologies and ecological buffer zone design methods to restore the site conditions necessary for their natural growth [105].

#### 5.1.4. Genetic Resources of Ancient Trees and Analysis of Longevity Mechanisms

With the rapid advancement of genomics and epigenetics, deciphering the molecular secrets behind the longevity and delayed senescence of ancient trees is becoming feasible. Future research requires a systematic analysis of the intrinsic genetic basis enabling ancient trees to exceed normal lifecycles, encompassing aspects such as genome stability, DNA damage repair, telomere dynamics, epigenetic modifications, and the evolution of gene families related to disease resistance. Utilizing ancient DNA technology, historical changes in population genetic structure can be traced from archival specimens, revealing genetic imprints of adaptation to past environmental fluctuations. Simultaneously, establishing a global germplasm gene bank and phenotypic database for ancient trees, alongside whole-genome resequencing of core germplasms, will facilitate the identification of key alleles associated with longevity and stress tolerance. This provides a foundation for preserving and utilizing the genetic resources of ancient trees through modern biotechnology [106]. Building upon this, systematic research on ancient tree conservation technologies urgently needs strengthening. In particular, comprehensive health diagnostic techniques should integrate molecular markers, physiological and biochemical indicators, and non-destructive testing to construct an early warning system.

#### 5.1.5. Digital Technology-Enabled Intelligent Monitoring and Conservation of Ancient Trees

Keywords such as “digital twin”, “machine learning” and “remote sensing” indicate growing interest in digital approaches to ancient tree research [63]. However, most applications remain pilot or model-based rather than large-scale deployments [105]. A few real-world deployments are emerging—for example, Guangzhou’s Huangpu Ancient Tree Park uses an intelligent monitoring network covering ancient trees with 10 s alerts and automated work orders, and Yongzhou’s “Ancient Tree Cloud Shield” combines IoT sensors and AI cameras to cut maintenance response from 24 h to under 5 min—but they remain site-specific pilots rather than large-scale practice. A potential future direction is an integrated “sky–air–ground” monitoring system, including LiDAR-equipped drones for 3D modeling and health inversion, IoT sensors for sap flow, stem growth, and soil moisture, and AI algorithms for pest and disease identification and decline risk warning. Multi-source data could be integrated on a digital twin platform to support virtual evaluation of conservation interventions, supporting more proactive and precise management. Yet, implementation faces bottlenecks: high equipment and maintenance costs, limited power and network coverage, inconsistent data standards, lack of long-term species-specific baseline data, poor AI model transferability, scarce labeled data, and barriers in data sharing, funding, and interdisciplinary capacity. Digital technologies should therefore complement, not replace, conventional conservation. The evaluation framework should also be more standardized, covering genetic integrity, physiological vitality, structural stability, habitat suitability, and ecosystem service function; combined with long-term in situ observation and big data analysis, it could help quantify intervention effectiveness and support refined management and emergency protection [2].

#### 5.1.6. Toward a Formal Strategy for International Collaboration in Ancient Tree Research

Our bibliometric results reveal that ancient tree research is geographically concentrated, with limited cross-border collaboration and a fragmented knowledge structure, as indicated by the zero betweenness centrality of highly cited works and a very low keyword network density. A coordinated international strategy is therefore needed. We propose four priorities: (1) establish a global consortium to harmonize definitions, indicators, and research priorities; (2) build a shared database and long-term monitoring network using FAIR principles and common protocols for tree-ring, genetic, microbial, and remote sensing data; (3) promote interdisciplinary and cross-regional collaboration, with capacity building and funding equity for underrepresented regions in Africa, South America, and Southeast Asia; and (4) align research with international policy frameworks such as the Kunming–Montreal Global Biodiversity Framework, UNFCCC, and UNESCO, and translate findings into conservation guidelines. Regular bibliometric assessments should monitor collaboration and adjust the strategy over time. This would move ancient tree research from fragmented national efforts toward a truly global and policy-relevant endeavor.

### 5.2. Research Limitations

While bibliometric analysis offers new insights into ancient tree research, its methodological limitations must be acknowledged. In particular, reliance on the Web of Science (WOS) introduces a coverage bias because WOS mainly indexes peer-reviewed journal articles and conference proceedings and excludes most gray literature, such as technical reports, policy documents, local management plans, unpublished surveys, and non-indexed regional journals. This omission may underrepresent on-the-ground conservation activities, local and Indigenous knowledge, and nuanced cultural dialogs that are often documented outside the formal academic literature. In addition, the merging and normalization of institution names in WOS can be imperfect: variant spellings, abbreviations, translated names, and subsidiary units may be split or conflated, potentially distorting institutional productivity, collaboration patterns, and comparative rankings. These data-selection and methodological constraints call for cautious interpretation of the findings. Future research could therefore combine big data approaches, such as meta-analysis and machine learning, with in-depth qualitative and contextual interpretation of both the indexed and gray literature.

## 6. Conclusions

Using CiteSpace 6.4.R1, this study analyzed 462 international publications on ancient trees from 1998 to 2025. The main conclusions are as follows. (1) Publication output and disciplinary coverage: Ancient tree research has shown a steady upward trend, moving from initial exploration to rapid development and accelerated growth. The field has expanded from forestry and plant sciences to ecology, environmental sciences, genetics, and microbiology, indicating a shift from single-process studies to interdisciplinary and systematic approaches. (2) Research entities and collaboration: China and the United States are the leading contributors, and the Chinese Academy of Sciences is among the most productive institutions. However, the generally low centrality of most institutions indicates limited international collaboration. Strengthening cross-border cooperation remains a key challenge. (3) Knowledge structure and fragmentation: Highly cited works have attracted sustained attention. Combined with the low network density, this suggests that citation flows are concentrated within separate subfields. Ancient tree research therefore lacks bridging studies that connect ecology, genetics, microbiology, climate science, and social science. (4) Research hotspots and trends: Keyword analysis shows that growth, biodiversity, conservation, diversity, and biomass remain core themes. Recent bursts of forest management, biodiversity, and climate indicate a shift toward management-oriented, biodiversity-centered, and climate-adaptive research. Future directions include social–ecological integration, climate response, microbial symbiosis, genetic resource conservation, and digital intelligent monitoring. Overall, ancient tree research is growing and diversifying, but it remains geographically concentrated and intellectually fragmented. These characteristics highlight the need for stronger international collaboration and more integrated research frameworks.

## Figures and Tables

**Table 1:** Most cited by the top 20 journals.

Rank	Number	Year	Journal Name	JCR Partitioning/IF in 2024	Journal Ranking
1	183	1999	*Forest Ecology and Management*	Q1; IF:3.7	10/92
2	150	2001	*Biological Conservation*	Q1; IF:4.4	8/74
3	120	2002	*Nature*	Q1; IF:48.5	2/136
4	118	1999	*Science*	Q1; IF:45.8	3/136
5	109	2013	*PLOS One*	Q2; IF:2.6	44/136
6	103	1999	*Ecology*	Q1; IF:4.3	32/200
7	88	2008	*PNAS*	Q1; IF:9.1	14/136
8	86	1998	*Canadian Journal of Forest Research*	Q2; IF:1.5	45/92
9	81	2003	*New Phytologist*	Q1; IF:8.1	12/273
10	71	1999	*Tree Physiology*	Q1; IF:3.7	10/92
12	66	2007	*Journal of Ecology*	Q1; IF:5.6	20/200
13	66	2008	*Biodiversity and Conservation*	Q1; IF:3.1	16/74
14	66	2016	*Forests*	Q2; IF:2.5	25/92
15	61	2017	*Science of The Total Environment*	Q1; IF:8.0	39/376
16	60	2008	*Global Change Biology*	Q1; IF:12.1	1/74
17	57	2013	*Trends in Ecology & Evolution*	Q1; IF:17.3	1/200
18	57	1999	*Oecologia*	Q2; IF:2.3	95/200
19	55	2008	*Ecological Applications*	Q1; IF:4.3	32/200
20	53	2008	*Ecology Letters*	Q1; IF:7.9	7/200

**Table 2:** Keywords highlighted.

Top 3 Keywords with the Strongest Citation Bursts					
Keywords	Year	Strength	Begin	End	1998–2025
forest management	2016	3.47	**2016**	2021	▂▂▂▂▂▂▂▂▂▂▂▂▂▂▂▂▂▂▃▃▃▃▃▃▂▂▂▂
biodiversity	2005	5.39	**2017**	2023	▂▂▂▂▂▂▂▂▂▂▂▂▂▂▂▂▂▂▂▃▃▃▃▃▃▃▂▂
climate	2019	3.85	**2019**	2025	▂▂▂▂▂▂▂▂▂▂▂▂▂▂▂▂▂▂▂▂▂▃▃▃▃▃▃▃

## Data Availability

No new data were created or analyzed in this study.

## Appendix A

**Table A1:** CiteSpace parameter settings and algorithm details.

Parameter	Setting/Algorithm	Definition and Role in This Study
Software	CiteSpace 6.4.R1	Visual bibliometric analysis software
Data source	Web of Science Core Collection	Search date: 15 November 2025
Document types	Article, Review, Proceedings Paper	English-language publications only
Final dataset	462 publications	1998–2025
Time span	January 1998–December 2025	Full analysis period
Time slice	1 year	Each year treated as one time slice
Node types	Country, Institution, Keyword	Used for collaboration and co-occurrence networks
Node selection threshold	g-index, *k* = 25	Applied to each time slice to select influential nodes
Auxiliary selection parameters	LRF = 3.0; L/N = 10; LBY = 8; e = 1.0	LRF: link retaining factor; L/N: links per node; LBY: look-back years; e: minimum occurrence in Top N mode
Link strength	Cosine	Calculates similarity between nodes
Pruning algorithm	Minimum Spanning Tree (MST) + Pruning Sliced Networks	Pruning Sliced Networks was applied to each time slice; MST retained the network backbone after merging
Centrality	Normalized betweenness centrality	$C_{B}(v)=\sum_{s\neq v\neq t}\frac{\sigma_{st}(v)}{\sigma_{st}}$; normalized to [0, 1]
High-centrality threshold	≥0.1	Used to identify key bridge nodes
Cluster labeling	Log-Likelihood Ratio (LLR)	Used for keyword and term clustering
Cluster evaluation	Modularity *Q*; Silhouette *S*	*Q* > 0.3 and *S* > 0.5 considered acceptable;
Burst detection	Kleinberg’s algorithm	Used to identify emerging keywords
Keyword co-occurrence network	N = 644, E = 1537, density = 0.0074	Reported in the keyword co-occurrence analysis

## References

[1] Xie C.P., Dong W.C., Liu D.W. (2020). Species diversity and distribution pattern of old trees in wuzhong district, suzhou city. Pak. J. Bot..

[2] Fu Q.C., Qiu E.F., Zhang Y., Huang L.H., Wang H.C., Jiang S.S. (2022). Discussion of the Distribution Pattern and Driving Factors of 2 Large Old Tree Resources in Beijing. Forests.

[3] Wang X., Han J., Liu P., Guo D., Jiang M. (2026). Human Activities and Climate Jointly Shape the Old-Tree Diversity in Human-Dominated Landscapes of the Yellow River Basin, China. Forests.

[4] Brown P.M., Gannon B., Battaglia M.A., Fornwalt P.J., Huckaby L.S., Cheng A.S., Baggett L.S. (2019). Identifying old trees to inform ecological restoration in montane forests of the central rocky mountains, USA. Tree-Ring Res..

[5] Wang X., Zhang Y., McRae D.J. (2009). Spatial and age-dependent tree-ring growth responses of Larix gmelinii to climate in northeastern China. Trees.

[6] Spornberger A., Noll D., Bodner P., Grall G. (2021). Recording and preservation of apple cultivars suitable for high altitudes in the region of Naturpark Otscher-Tormauer in Lower Austria. Mitteilungen Klosterneubg. Rebe Wein Obstbau Früchteverwert..

[7] Yang B., Qin C., Bräuning A., Osborn T.J., Trouet V., Ljungqvist F.C., Esper J., Schneider L., Grießinger J., Büntgen U. (2021). Long-term decrease in Asian monsoon rainfall and abrupt climate change events over the past 6,700 years. Proc. Natl. Acad. Sci. USA.

[8] Björklund J., Seftigen K., Stoffel M., Fonti M.V., Kottlow S., Frank D.C., Esper J., Fonti P., Goosse H., Grudd H. (2023). Fennoscandian tree-ring anatomy shows a warmer modern than medieval climate. Nature.

[9] Huang L., Tian L.J., Zhou L.H., Jin C., Qian S.H., Jim C.Y., Lin D.M., Zhao L., Minor J., Coggins C. (2020). Local cultural beliefs and practices promote conservation of large old trees in an ethnic minority region in southwestern China. Urban For. Urban Green..

[10] Lindenmayer D.B., Laurance W.F., Franklin J.F. (2012). Global Decline in Large Old Trees. Science.

[11] Qin C., Ma J., Liu J. (2025). Current situation, problems and suggestions of ancient and notable tree resources in Gansu Province China. Chin. J. Appl. Ecol..

[12] Mu Y.M., Lindenmayer D., Zheng S.L., Yang Y.C., Wang D.Y., Liu J.J. (2023). Size-focused conservation may fail to protect the world’s oldest trees. Curr. Biol..

[13] Liu J.J., Xia S.W., Zeng D., Liu C., Li Y.J., Yang W.J., Yang B., Zhang J., Slik F., Lindenmayer D.B. (2022). Age and spatial distribution of the world’s oldest trees. Conserv. Biol..

[14] Johnston J.D., Stephens P.N., Schmidt M.R., Jeronimo S., Brackett A.E., Wuenschel A.E., Ulrich D., Paine R.H., Duloisy L.F., Greenler S.M. (2025). Significant mortality of old trees across a dry forest landscape, Oregon, USA. For. Ecol. Manag..

[15] Lindenmayer D.B., Laurance W.F. (2016). The Unique Challenges of Conserving Large Old Trees. Trends Ecol. Evol..

[16] Chen Y., He F., Han Y. (2023). Present situation and spatial layout of ancient and famous trees in Nanjing. J. Cent. South Univ. For. Technol..

[17] Piovesan G., Cannon C.H., Liu J.J., Munné-Bosch S. (2022). Ancient trees: Irreplaceable conservation resource for ecosystem restoration. Trends Ecol. Evol..

[18] Liu J.J., Lindenmayer D.B., Yang W.J., Ren Y., Campbell M.J., Wu C.P., Luo Y.Q., Zhong L., Yu M.J. (2019). Diversity and density patterns of large old trees in China. Sci. Total Environ..

[19] Jim C.Y., Zhang H. (2013). Species diversity and spatial differentiation of old-valuable trees in urban Hong Kong. Urban For. Urban Green..

[20] Xie C.P., Huang C., Jiang S.S., Tian E., Liu D.W. (2025). Heritage Trees of Lingshui, China: Diversity, Distribution, and Conservation Challenges. Bioresources.

[21] Zhou L.B., Zhang Q. (2022). Multiple indicators metrological analysis for 5 kinds of tea produced in Yunnan, China. Food Sci. Technol..

[22] Lindenmayer D.B., Blanchard W., Blair D., McBurney L., Banks S.C. (2016). Environmental and human drivers influencing large old tree abundance in Australian wet forests. For. Ecol. Manag..

[23] Sabe M., Pillinger T., Kaiser S., Chen C.M., Taipale H., Tanskanen A., Tiihonen J., Leucht S., Correll C.U., Solmi M. (2022). Half a century of research on antipsychotics and schizophrenia: A scientometric study of hotspots, nodes, bursts, and trends. Neurosci. Biobehav. Rev..

[24] Pan X.L., Yan E.J., Cui M., Hua W.N. (2018). Examining the usage, citation, and diffusion patterns of bibliometric mapping software: A comparative study of three tools. J. Informetr..

[25] Xie C., Liu C., Liu D., Jim C.Y. (2024). Charting the Research Terrain for Large Old Trees: Findings from a Quantitative Bibliometric Examination in the Twenty-First Century. Forests.

[26] Ding X., Yang Z. (2022). Knowledge mapping of platform research: A visual analysis using VOSviewer and CiteSpace. Electron. Commer. Res..

[27] Zhang S., Wen L.S., Weng A.F., Cheng D.L., Li B.Y. (2024). Research Progress in Biodiversity and Human Well-Being, Based on CiteSpace. Biology.

[28] Huang L., Tian L., Huang L., Jin C., Hu S., Zhang Z., Yan E., Jim C.Y., Yang Y., Lindenmayer D.B. (2025). Religious temples are long-term refuges for old trees in human-dominated landscapes in China. Curr. Biol..

[29] Huang L., Jin C., Pan Y., Zhou L., Hu S., Guo Y., Meng Y., Song K., Pang M., Li H. (2023). Human activities and species biological traits drive the long-term persistence of old trees in human-dominated landscapes. Nat. Plants.

[30] Zhou X.Q., Guo Z.Y., Chen C.R., Jia Z.J. (2017). Soil microbial community structure and diversity are largely influenced by soil pH and nutrient quality in 78-year-old tree plantations. Biogeosciences.

[31] Phillips N.G., Buckley T.N., Tissue D.T. (2008). Capacity of Old Trees to Respond to Environmental Change. J. Integr. Plant Biol..

[32] Kuuluvainen T., Gauthier S. (2018). Young and old forest in the boreal: Critical stages of ecosystem dynamics and management under global change. For. Ecosyst..

[33] Pasques O., Munné-Bosch S. (2024). Ancient trees are essential elements for high-mountain forest conservation: Linking the longevity of trees to their ecological function. Proc. Natl. Acad. Sci. USA.

[34] Liu S., Xu H., Wang G., Jin B., Cao F., Wang L. (2024). Tree Longevity: Multifaceted Genetic Strategies and Beyond. Plant Cell Environ..

[35] Piovesan G., Biondi F. (2021). On tree longevity. New Phytol..

[36] Chen Y., Huang H., Li J., Zheng Z., Gao F., Han X., Gao Y. (2025). Digital twin comprehensive models: A study of ancient tree ecological environment quality assessment based on a cyber-physical system. Environ. Monit. Assess..

[37] Dinca L., Constandache C., Murariu G., Antofie M.M., Draghici T., Bratu I. (2025). Environmental Archaeology Through Tree Rings: Dendrochronology as a Tool for Reconstructing Ancient Human–Environment Interactions. Heritage.

[38] Ibarra J.T., Riquelme-Maulén W., Bañales-Seguel C., Orrego G., Salazar G. (2024). While clearing the forests: The social–ecological memory of trees in the Anthropocene. Ambio.

[39] Li J., Liu Y., Wei Y., Li J., Zhang K., Wei X., Peng J. (2024). Dendroclimatological study of ancient trees integrating non-destructive techniques. Front. Plant Sci..

[40] Guo K., Guo J., Gong X., Shi H., Zhou Y., Leng Q., Hao G. (2024). Coordinated responses of Hemiptelea davidii at the individual tree and stand levels to interannual climatic variation in a water-limited area. For. Ecol. Manag..

[41] Wieland A., Römer P., Torbenson M., Greule M., Urban O., Čáslavský J., Pernicová N., Trnka M., Büntgen U., Esper J. (2024). Tree-ring stable isotopes in cellulose and lignin methoxy groups reveal different age-related behaviour. Quat. Int..

[42] Italiano S.S.P., Camarero J.J., Colangelo M., Borghetti M., Castellaneta M., Pizarro M., Ripullone F. (2023). Assessing Forest Vulnerability to Climate Change Combining Remote Sensing and Tree-Ring Data: Issues, Needs and Avenues. Forests.

[43] Altman J., Altmanova N., Fibich P., Korznikov K., Fonti P. (2025). Advancing dendrochronology with R: An overview of packages and future perspectives. Front. Ecol. Evol..

[44] Zhang S., Liang J., Su X., Chen Y., Wei Q. (2023). Research on global cultural heritage tourism based on bibliometric analysis. Herit. Sci..

[45] Chen C.M. (2006). CiteSpace II: Detecting and visualizing emerging trends and transient patterns in scientific literature. J. Am. Soc. Inf. Sci. Technol..

[46] Chen C.M., Song I.Y., Yuan X.J., Zhang J. (2008). The thematic and citation landscape of Data and Knowledge Engineering (1985–2007). Data Knowl. Eng..

[47] Uredništvo HŠD (2023). On the occasion of march 21st, international day of forests. Šumar. List.

[48] Niu L., Zhao X., Wu F., Tang Z., Lv H., Wang J., Fang M., Giesy J.P. (2021). Hotpots and trends of covalent organic frameworks (COFs) in the environmental and energy field: Bibliometric analysis. Sci. Total Environ..

[49] Xiao C., Hu Q., Chen X., Zhao T., Guo X., Chen F. (2023). Research Progress of nitrogen deposition based on bibliometrics. Acta Ecol. Sin..

[50] Cannon C.H., Piovesan G., Munné-Bosch S. (2022). Old and ancient trees are life history lottery winners and vital evolutionary resources for long-term adaptive capacity. Nat. Plants.

[51] Gilhen-Baker M., Roviello V., Beresford-Kroeger D., Roviello G.N. (2022). Old growth forests and large old trees as critical organisms connecting ecosystems and human health. A review. Environ. Chem. Lett..

[52] Lindenmayer D.B., Laurance W.F. (2017). The ecology, distribution, conservation and management of large old trees. Biol. Rev..

[53] Nolan V., Reader T., Gilbert F., Atkinson N. (2020). The Ancient Tree Inventory: A summary of the results of a 15 year citizen science project recording ancient, veteran and notable trees across the UK. Biodivers. Conserv..

[54] Zhang H., Lai P.Y., Jim C.Y. (2017). Species diversity and spatial pattern of old and precious trees in Macau. Landsc. Urban Plan..

[55] Adabre M.A., Chan A., Darko A. (2021). A scientometric analysis of the housing affordability literature. J. Hous. Built Environ..

[56] Li Y., Huang H., Wang S. (2023). Hotspots and trends of rural resilience research: Knowledge map analysis based on CiteSpace. Sci. Geogr. Sin..

[57] Chen C. (2017). Science Mapping: A Systematic Review of the Literature. J. Data Inf. Sci..

[58] Amon C., Dyderski M.K., Oettel J., Schueler S., Essl F., Lapin K. (2025). Differences in tree diversity, structural diversity, and aboveground carbon storage across Austrian forest types—Implications for forest conservation management. For. Ecol. Manag..

[59] Wessely J., Essl F., Fiedler K., Gattringer A., Hülber B., Ignateva O., Moser D., Rammer W., Dullinger S., Seidl R. (2024). A climate-induced tree species bottleneck for forest management in Europe. Nat. Ecol. Evol..

[60] Niang F., Marchand P., Sambou B., Fenton N. (2024). Exploring the effects of forest management on tree diversity, community composition, population structure and carbon stocks in sudanian domain of Senegal, West Africa. For. Ecol. Manag..

[61] Wan J.Z., Li Q.F., Wei G.L., Yin G.J., Wei D.X., Song Z.M., Wang C.J. (2020). The effects of the human footprint and soil properties on the habitat suitability of large old trees in alpine urban and periurban areas. Urban For. Urban Green..

[62] Kebrle D., Zasadil P., Hošek J., Barták V., Šťastný K. (2021). Large trees as a key factor for bird diversity in spruce-dominated production forests: Implications for conservation management. For. Ecol. Manag..

[63] Moosavi S.J., Mueller M., Gailing O. (2025). Development of New Chloroplast Microsatellites for *Pinus gerardiana* and their Application in Genetic Diversity Analyses. Ecol. Evol..

[64] Zhou H., Zhang X., Liu H., Ma J., Hao F., Ye H., Wang Y., Zhang S., Yue M., Zhao P. (2024). Chromosome-level genome assembly of *Platycarya strobilacea*. Sci. Data.

[65] Martijn J., Lind A.E., Spiers I., Juzokaite L., Bunikis I., Vinnere Pettersson O., Ettema T.J.G. (2017). Amplicon sequencing of the 16S-ITS-23S rRNA operon with long-read technology for improved phylogenetic classification of uncultured prokaryotes. bioRxiv.

[66] Stahle D.W. (2022). Natural monuments Reverence for old trees sparked early forest conservation efforts, even as ancient woodlands were cut with impunity. Science.

[67] Wahab A., Muhammad M., Munir A., Abdi G., Zaman W., Ayaz A., Khizar C., Reddy S.P.P. (2023). Role of Arbuscular Mycorrhizal Fungi in Regulating Growth, Enhancing Productivity, and Potentially Influencing Ecosystems under Abiotic and Biotic Stresses. Plants.

[68] Busso M.A., Busso M. (2022). Arbuscular mycorrhizal fungi and common mycorrhizal networks benefit plants through morphological, physiological and productive traits and soil quality. Lilloa.

[69] Xu C., Huang R., An W., Zhao Q., Zhao Y., Ren J., Liu Y., Guo Z. (2024). Tree ring oxygen isotope in Asia. Glob. Planet. Change.

[70] Langor D.W., Hammond H.E.J., Spence J.R., Jacobs J., Cobb T.P. (2008). Saproxylic insect assemblages in Canadian forests: Diversity, ecology, and conservation. Can. Entomol..

[71] Fang J., Chen A., Peng C., Zhao S., Ci L. (2001). Changes in Forest Biomass Carbon Storage in China Between 1949 and 1998. Science.

[72] Nave L.E., Walters B.F., Hofmeister K.L., Perry C.H., Mishra U., Domke G.M., Swanston C.W. (2018). The role of reforestation in carbon sequestration. New For..

[73] Oleribe O., Miller R., Wadzeck M., Mendez N., Tibay J., Langford T., Devine A., Taylor-Robinson S.D. (2021). Klamath Tribal Response to the Pandemic of COVID-19 Among Klamath Tribal Community in Oregon, USA. Glob. Adv. Health Med..

[74] Johnston J.D., Greenler S.M., Reilly M.J., Webb M.R., Merschel A.G., Johnson K.N., Franklin J.F. (2021). Conservation of Dry Forest Old Growth in Eastern Oregon. J. For..

[75] Trlica A., Hutyra L.R., Morreale L.L., Smith I.A., Reinmann A.B. (2020). Current and future biomass carbon uptake in Boston’s urban forest. Sci. Total Environ..

[76] Bhagwat S.A., Nogué S., Willis K.J. (2012). Resilience of an ancient tropical forest landscape to 7500 years of environmental change. Biol. Conserv..

[77] Akhbarfar G., Nikbakht A., Etemadi N., Gailing O. (2023). Physiological and Biochemical Responses of Plantain Trees (*Platanus orientalis* L.) Derived from Different Ages to Drought Stress and *Ascophyllum nodosum* L. Extract. J. Soil Sci. Plant Nutr..

[78] Ballester A., San-José M.C., Vidal N., Fernández-Lorenzo J.L., Vietez A.M. (1999). Anatomical and biochemical events during in vitro rooting of microcuttings from juvenile and mature phases of chestnut. Ann. Bot..

[79] Wilkaniec A., Borowiak-Sobkowiak B., Irzykowska L., Bres W., Swierk D., Pardela L., Durak R., Srodulska-Wielgus J., Wielgus K. (2021). Biotic and abiotic factors causing the collapse of *Robinia pseudoacacia* L. veteran trees in urban environments. PLoS ONE.

[80] Chen C.M. (2004). Searching for intellectual turning points: Progressive knowledge domain visualization. Proc. Natl. Acad. Sci. USA.

[81] Toraño Caicoya A., Vergarechea M., Blattert C., Klein J., Eyvindson K., Burgas D., Snäll T., Mönkkönen M., Astrup R., Di Fulvio F. (2023). What drives forest multifunctionality in central and northern Europe? Exploring the interplay of management, climate, and policies. Ecosyst. Serv..

[82] Shipley J.R., Gossner M.M., Rigling A., Krumm F. (2023). Conserving forest insect biodiversity requires the protection of key habitat features. Trends Ecol. Evol..

[83] Azuero-Pedraza C.G., Lauri P., Lessa Derci Augustynczik A., Thomas V.M. (2024). Managing Forests for Biodiversity Conservation and Climate Change Mitigation. Environ. Sci. Technol..

[84] Leclerc L., Calderón-Sanou I., Martinez-Almoyna C., Paillet Y., Thuiller W., Vincenot L., Kunstler G. (2023). Beyond the role of climate and soil conditions: Living and dead trees matter for soil biodiversity in mountain forests. Soil Biol. Biochem..

[85] Feng J., Dan X., Cui Y., Gong Y., Peng M., Sang Y., Ingvarsson P.K., Wang J. (2024). Integrating evolutionary genomics of forest trees to inform future tree breeding amid rapid climate change. Plant Commun..

[86] Potterf M., Eyvindson K., Blattert C., Triviño M., Burner R.C., Burgas D., Mönkkönen M. (2023). Diversification of forest management can mitigate wind damage risk and maintain biodiversity. Eur. J. For. Res..

[87] Nasielski J., Furze J.R., Tan J., Bargaz A., Thevathasan N.V., Isaac M.E. (2015). Agroforestry promotes soybean yield stability and N2-fixation under water stress. Agron. Sustain. Dev..

[88] Xie C.P., Jiang S.S., Liu D.W., Jim C.Y. (2025). Degrading Large Old Tree Population Deserves More Conservation Effort: A Case of Endangered Barringtonia racemosa in Tropical Rural China. Bioresources.

[89] Zhang S., Liu C.F., Chen Y.C., Liang J.X., Ma Y.Q. (2025). Research on the Impact of Climate Change Perceptions on the Carbon Offset Behavior of Visitors to Wuyi Mountain Forestry Heritage Site. Forests.

[90] Xing Z., Wu L., Biere A., Yu H., Ding J. (2025). Foliar Herbivory Suppresses Arbuscular Mycorrhizal Colonisation by Weakening Symbiosis Signalling in Root Exudates. Plant Cell Environ..

[91] Tong X., Brandt M., Yue Y., Ciais P., Rudbeck Jepsen M., Penuelas J., Wigneron J., Xiao X., Song X., Horion S. (2020). Forest management in southern China generates short term extensive carbon sequestration. Nat. Commun..

[92] Cantarello E., Jacobsen J.B., Lloret F., Lindner M. (2024). Shaping and enhancing resilient forests for a resilient society. Ambio.

[93] Radies D., Coxson D., Johnson C., Konwicki K. (2009). Predicting canopy macrolichen diversity and abundance within old-growth inland temperate rainforests. For. Ecol. Manag..

[94] Liu F., Liu H., Xu C., Shi L., Zhu X., Qi Y., He W. (2021). Old-growth forests show low canopy resilience to droughts at the southern edge of the taiga. Glob. Change Biol..

[95] LaRue E.A., Atkins J.W., Dahlin K., Fahey R., Fei S., Gough C., Hardiman B.S. (2018). Linking Landsat to terrestrial LiDAR: Vegetation metrics of forest greenness are correlated with canopy structural complexity. Int. J. Appl. Earth Obs..

[96] Manabe T., Shimatani K., Kawarasaki S., Aikawa S.I., Yamamoto S.I. (2008). The patch mosaic of an old-growth warm-temperate forest: Patch-level descriptions of 40-year gap-forming processes and community structures. Ecol. Res..

[97] Mugnani M.P., Robertson K.M., Miller D.L., Platt W.J. (2019). Longleaf Pine Patch Dynamics Influence Ground-Layer Vegetation in Old-Growth Pine Savanna. Forests.

[98] Reiley D.K., Breshears D.D., Zedler P.H., Ebinger M.H., Meyer C.W. (2010). Soil carbon heterogeneity in pinon–juniper woodland patches: Effect of woody plant variation on neighboring intercanopies is not detectable. J. Arid Environ..

[99] Segoli M., Ungar E.D., Shachak M. (2008). Shrubs enhance resilience of a semi-arid ecosystem by engineering and regrowth. Ecohydrology.

[100] Xu C., Liu H., Anenkhonov O.A., Korolyuk A.Y., Sandanov D.V., Balsanova L.D., Naidanov B.B., Wu X. (2016). Long-term forest resilience to climate change indicated by mortality, regeneration, and growth in semiarid southern Siberia. Glob. Change Biol..

[101] Xie C.P., Jim C.Y. (2025). Where culture merges with conservation: Indigenous wisdom preserving Jingmai’s ancient tea forests and heritage trees. Agrofor. Syst..

[102] Zhang S.B.W., Xu C.B., Wen L.S., Zhong Q.L., Hu Q.P., Li B.Y., Chen B.B. (2026). Spatiotemporal patterns and carbon balance zoning optimization of county-level carbon budget in Fujian Province. iScience.

[103] Luo Y., Chen H. (2013). Observations from old forests underestimate climate change effects on tree mortality. Nat. Commun..

[104] Shive K.L., Wuenschel A., Hardlund L.J., Morris S., Meyer M.D., Hood S.M. (2022). Ancient trees and modern wildfires: Declining resilience to wildfire in the highly fire-adapted giant sequoia. For. Ecol. Manag..

[105] Roccaforte J.P., Huffman D.W., Rodman K.C., Crouse J.E., Pedersen R.J., Normandin D.P., Fulé P.Z. (2024). Long-term ecological responses to landscape-scale restoration in a western United States dry forest. Restor. Ecol..

[106] Zheng J.M., Chen Y.S., Guan L., Ke K., Zhang H.R., Zhang J.X., Xiao Y. (2025). Predicting the age of ancient trees and the factors influencing their growth of Lychee trees (*Litchi chinensis*). npj Herit. Sci..

